# Phosphorylation of an RNA‐Binding Protein Rck/Me31b by Hippo Is Essential for Adipose Tissue Aging

**DOI:** 10.1111/acel.70022

**Published:** 2025-03-11

**Authors:** Eunbyul Yeom, Hyejin Mun, Jinhwan Lim, Yoo Lim Chun, Kyung‐Won Min, Johana Lambert, L. Ashley Cowart, Jason S. Pierce, Besim Ogretmen, Jung‐Hyun Cho, Jeong Ho Chang, J. Ross Buchan, Jason Pitt, Matt Kaeberlein, Sung‐Ung Kang, Eun‐Soo Kwon, Seungbeom Ko, Kyoung‐Min Choi, Yong Sun Lee, Yoon‐Su Ha, Seung‐Jin Kim, Kwang‐Pyo Lee, Hyo‐Sung Kim, Seo Young Yang, Chang Hoon Shin, Je‐Hyun Yoon, Kyu‐Sun Lee

**Affiliations:** ^1^ School of Life Sciences, BK21 FOUR KNU Creative BioResearch Group Kyungpook National University Daegu Korea; ^2^ KNU G‐LAMP Project Group, KNU Institute of Basic Sciences, School of Life Sciences, College of Natural Sciences Kyungpook National University Daegu Korea; ^3^ Neurophysiology and Metabolism Research Group Korea Research Institute of Bioscience and Biotechnology (KRIBB) Daejeon Korea; ^4^ Department of Biochemistry and Molecular Biology Medical University of South Carolina Charleston South Carolina USA; ^5^ Department of Oncology Science University of Oklahoma Oklahoma City Oklahoma USA; ^6^ Department of Environmental and Occupational Heatlh University of California Irvine California USA; ^7^ Translational Gerontology Branch National Institute of Aging Intramural Research Program Baltimore Maryland USA; ^8^ Department of Biology, College of Natural Sciences Gangneung‐Wonju National University Gangneung South Korea; ^9^ Department of Biochemistry and Molecular Biology and the Massey Cancer Center Virginia Commonwealth University Richmond Virginia USA; ^10^ Hunter Holmes McGuire Veteran's Affairs Medical Center Richmond Virginia USA; ^11^ Hollings Cancer Center Medical University of South Carolina Charleston South Carolina USA; ^12^ Department of Biology Education Kyungpook National University Daegu Republic of Korea; ^13^ Department of Molecular and Cellular Biology University of Arizona Tucson Arizona USA; ^14^ Department of Laboratory Medicine and Pathology University of Washington Washington DC USA; ^15^ Neuroregeneration and Stem Cell Programs Institute for Cell Engineering Baltimore Maryland USA; ^16^ Department of Neurology Johns Hopkins University School of Medicine Baltimore Maryland USA; ^17^ Aging Research Center Korea Research Institute of Bioscience and Biotechnology (KRIBB) Daejeon Korea; ^18^ Department of Cancer Biomedical Science, Graduate School of Cancer Science and Policy National Cancer Center Goyang Republic of Korea; ^19^ Department of Biochemistry, College of Natural Sciences Kangwon National University Chuncheon Republic of Korea; ^20^ Department of Pathology University of Oklahoma Oklahoma City Oklahoma USA; ^21^ School of Pharmacy Sungkyunkwan University Suwon Korea

**Keywords:** adipocyte, differentiation, fat body, hippo, lifespan, lipid metabolism, mRNA decay

## Abstract

The metazoan lifespan is determined in part by a complex signaling network that regulates energy metabolism and stress responses. Key signaling hubs in this network include insulin/IGF‐1, AMPK, mTOR, and sirtuins. The Hippo/Mammalian Ste20‐like Kinase1 (MST1) pathway has been reported to maintain lifespan in *Caenorhabditis elegans* , but its role has not been studied in higher metazoans. In this study, we report that overexpression of Hpo, the MST1 homolog in *Drosophila melanogaster* , decreased lifespan with concomitant changes in lipid metabolism and aging‐associated gene expression, while RNAi Hpo depletion increased lifespan. These effects were mediated primarily by Hpo‐induced transcriptional activation of the RNA‐binding protein maternal expression at 31B (Me31b)/RCK, resulting in stabilization of mRNA‐encoding a lipolytic hormone, Akh. In mouse adipocytes, Hpo/Mst1 mediated adipocyte differentiation, phosphorylation of RNA‐binding proteins such as Rck, decapping MRNA 2 (Dcp2), enhancer Of MRNA decapping 3 (Edc3), nucleolin (NCL), and *glucagon* mRNA stability by interacting with Rck. Decreased lifespan in Hpo‐overexpressing *Drosophila* lines required expression of Me31b, but not DCP2, which was potentially mediated by recovering expression of lipid metabolic genes and formation of lipid droplets. Taken together, our findings suggest that Hpo/Mst1 plays a conserved role in longevity by regulating adipogenesis and fatty acid metabolism.

## Graphical abstract

Evolutionary conserved Hippo/Mst1 signaling non‐canonically regulates the stability of mRNAs through directly phosphorylating a subset of RNA binding proteins to stabilize lipolytic mRNAs. Hpo pathway contributes to the regulation of Drosophila lifespan by modulating the expression and activity of Me31b/RCK, which subsequently affects mRNA levels of adipogenic or lipogenic genes.

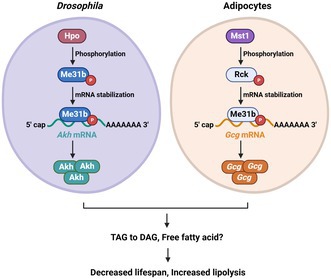

## 1 Introduction

Aging is defined as time‐dependent changes in physiology and accumulation of pathologies and affects most living organisms (López‐Otín et al. 2013). Aging includes declined metabolic efficiency, accumulation of damage within cells and loss of homeostatic capacity, including pH homeostasis (Wiley and Campisi 2016). Signaling pathways with known roles in metazoan lifespan have been studied extensively and include the well‐characterized insulin/Insulin‐like growth factor‐1 (IGF‐1) pathway, Mammalian target of rapamycin (mTOR) signaling, and sirtuin pathways (Houtkooper et al. 2012; Mori et al. 2014; Van Heemst 2010). For example, insulin/IGF‐1 peptides are recognized by their membrane receptors, activating a downstream relay of phosphorylation for the transcriptional activation and repression of target genes (Van Heemst 2010), and the suppression of insulin/IGF‐1 signaling increases lifespan in *C. elegans* (Gems et al. 1998; Tissenbaum and Ruvkun 1998; Lee and Lee 2022), *Drosophila melanogaster* (Tatar et al. 2001), and mice (Blüher et al. 2003). Similarly, the mechanistic target of rapamycin (mTOR) is a kinase that responds to nutrient availability to regulate lifespan through phosphorylation of target genes related to mRNA translation, autophagy, and several other processes (Kaeberlein 2013). Both insulin/IGF‐1 signaling and mTOR interact in a network with key metabolic regulators such as AMP‐activated protein kinase (AMPK) and sirtuins to orchestrate signaling cascades and energy metabolism and regulate metazoan lifespan.

Additional signaling pathways involved in determining the metazoan lifespan include the canonical mitogen‐activated protein kinase (MAPK) pathways comprised of MST1 and its downstream kinases MAPK kinase 4/7 (MKK4/7) and c‐Jun N‐terminal Kinase (JNK), which play well‐characterized roles in stress responses (Biteau et al. 2011; Lehtinen et al. 2006). The *C. elegans* MST1 homolog, serine/threonine‐protein kinase‐1 (cst‐1) targets dauer formation‐16 (DAF‐16), and the *Drosophila* JNK homolog dJNK also regulates the class O of forkhead box transcription factors (dFoxo) activity to modulate lifespan (Lehtinen et al. 2006). A recent report also revealed that mammalian MST1 directly phosphorylates eukaryotic translation initiation factor 4e (eIF4E) at the threonine 55 residue to inhibit its ability to bind the 5′ 7‐methylguanosine CAP (5′‐CAP) and suppress mRNA translation, suggesting alternative pathways for lifespan control via modulation of mRNA translation (Min et al. 2017).

Metazoan longevity and age‐related diseases are regulated in part by adipose tissue, which contributes to lipid storage, metabolism, inflammation, and whole‐body energy homeostasis (Guo and Chumlea 1999; Luo and Liu 2016; Lutz and Woods 2012). Adipocyte proliferation and differentiation are key regulators of cell growth and longevity, which are modulated by Hippo/Mst1 signaling in *Drosophila* (Zheng and Pan 2019). In mammals, MST1 overexpression impairs insulin secretion by phosphorylating and destabilizing the β‐cell transcription factor pancreatic/duodenal homeobox‐1 (PDX1) (Ardestani et al. 2014). The MST2–Salvador 1 (SAV1) complex also promotes adipocyte differentiation by activating Peroxisome proliferators–activated receptor γ (PPARγ) (Park et al. 2012). Although adipose tissue abnormalities accelerate multiple age‐related diseases and impair longevity, no prior studies have assessed the potential relationship between Hippo/MST1 pathway regulation of adipogenic lipid metabolism and longevity.

In the present study, we identified that overexpression of MST1, the *Drosophila* Hpo homolog, decreases lifespan and promotes adipose tissue lipolysis. We found that MST1 directly phosphorylates the amino terminal (N‐terminal) threonine residues of RCK, stabilizing *Gcg* mRNA in mature mouse adipocytes. *Akh/Gcg* mRNAs interacted with Me31b/RCK in both *Akh* mRNA pull‐down and RCK immunoprecipitation studies. Our findings reveal the newly discovered and critical insight that the Hippo/MST1 pathway modulates adipogenic lipid metabolism and metazoan lifespan.

## 2 Materials and Methods

### 2.1 Cell Lines

Mouse 3 T3‐L1 and *Drosophila* S2 cells were obtained from ATCC (Manassas, VA). All cell lines were cultured in the media suggested by the suppliers, and the cell lines were independently validated by short tandem repeat DNA fingerprinting and chromosomal analysis in the Analytical Cell Models Core of the Medical University of South Carolina.

For differentiation of 3 T3‐L1 cells, 70% confluent cells were grown with MDI induction medium (0.5 mM IBMX, 1 μM dexamethasone, 10 μg/mL insulin) for 3 days. The culture media were then replaced with DMEM containing 10 μg/mL insulin, and cells were cultured for an additional 3 days. On day 6, the media were replaced with DMEM without insulin, and cells were grown for 1–4 subsequent days to induce differentiation into adipocyte‐like cells. *Drosophila* Hpo‐myc cDNA plasmid was provided by Dr. D.J. Pan and transfected into S2 cells using lipofectamine. Mouse Mst1 and Rck shRNAs were provided by Drs. D.S. Lim and E.J. Choi. The lentiviral vector pLV[shRNA]‐EGFP:T2A:Puro‐U6 > mStk4 and control plasmids were purchased from VectorBuilder (Chicago, USA).

Oil Red O staining was performed to verify lipid accumulation in 3T3‐L1 adipocytes. 3T3‐L1 pre‐adipocytes were induced to adipogenesis for 8 days in 12‐well plates. The cultured cells were then washed with phosphate‐buffered saline (PBS), fixed in 4% paraformaldehyde for 30 min at 4°C. Afterward, the cells were incubated in 60% isopropanol for 5 min and air‐dried. Subsequently, the cells were stained for 30 min with Oil Red O staining solution (Sigma‐Aldrich) and washed four times with distilled water. The stained lipid droplets were eluted with 100% isopropanol for 15 min. The amount of lipid droplet was quantified by measuring the absorbance at a wavelength of 520 nm in a microplate reader.

For isolation of primary murine adipose‐derived stem cells (ADSCs), all animal experiments conformed to the Guide for the Care and Use of Laboratory Animals and were in accordance with Public Health Service/National Institutes of Health guidelines for laboratory animal usage. Inguinal and axial subcutaneous fat pads were excised from 3 to 6‐week‐old male C57BL/6J mice (Jax #000664) and rinsed in 1× phosphate‐buffered saline with 1× antibiotic anti‐mycotic solution (Millipore Sigma A5955). Tissue was transferred to digestion buffer (100 mM HEPES, 120 mM NaCl, 50 nM KCl, 5 mM glucose, 1 mM CaCl_2_, 0.1% collagenase, 1.5% bovine serum albumin) and minced into small pieces. Minced tissue was then transferred to a 50 mL conical tube with a 25 mL serological pipette. Tissue was incubated at 37°C in a shaker at 150 rpm for 30 min, with manual shaking and observation every 10 min until digested. The digest was then filtered through a 100 μm cell strainer into a new 50 mL tube, diluted twofold with expansion medium (DMEM/F12 with 10% FBS and 1× antibiotic anti‐mycotic) and centrifuged at 500 *g* for 5 min. Floating lipid and media were aspirated, preserving only the cell pellet. The pellet was re‐suspended in expansion medium, filtered through a 40 μm cell strainer, and plated onto a tissue culture flask. Media was changed after 2 h to remove non‐adherent cells and debris. ADSCs were maintained at 37°C and 10% CO_2_ in expansion medium (changed every 2–3 days). Cells nearing confluency were split and re‐plated at 10,000 cells/cm^2^ on 6‐well culture plates for adipogenesis experiments.

### 2.2 *Drosophila* Genetics and Transgenes

*Drosophila melanogaster* was maintained at 25°C on standard media. *w* ^*1118*^ was used as wild‐type, and all stocks, including RNAi lines, were obtained from the Bloomington Stock Center (Bloomington, IN, USA). *UAS‐Hpo‐myc* was a gift from Dr. D.J. Pan. Fly stocks were maintained on standard cornmeal/agar (1.5%) medium at 25°C ± 1°C, 60% ± 5% humidity, and a 12:12 h light/dark cycle (Ashburner 1989). Wild‐type strains, *w* ^*1118*^ and eIF4E line (w^*1118*^; P{w[+mC] = UAS‐eIF4E1.A}26) were obtained from the Bloomington *Drosophila* Stock Center (Bloomington, Indiana). *UAS‐Hpo‐myc* was a gift from Dr. D.J. Pan. The RU486 inducible gene‐switch line, DA‐GSG, was kindly provided by V. Monnier. For the RU486 diet, the final concentration of RU486 was 200 μM, while the control diet contained equal amounts of ethanol.

### 2.3 *Drosophila* Lifespan Assay (Ubiquitous Expression)

Experimental fly (DA‐GSG, UAS‐eIF4E and UAS‐hpo) stocks were backcrossed with *w* ^*1118*^ more than five times and maintained as heterozygous lines, w^*1118*^; DA‐GSG/+, w^*1118*^; UAS‐*eIF4E*/+, and w^*1118*^; UAS‐*hpo*/+, respectively, before being used in lifespan assays. Two types of isogenic progeny, w^*1118*^; +/+ and w^*1118*^; DA‐GSG/DA‐GGS were collected from DA‐GSG/+ for subsequent crosses to minimize the effect of genetic background on lifespan. To obtain the Hpo RNAi knockdown or overexpression flies, homozygous UAS‐hpo RNAi and UAS‐hpo males were mated with w^*1118*^; DA‐GSG/DA‐GSG virgin females on the cornmeal food, respectively. UAS‐hpo RNAi and UAS‐hpo males were mated with w^*1118*^; +/+ virgin females to collect progeny UAS‐hpo RNAi/+ and UAS‐hpo/+ as the corresponding wild‐type controls. To obtain eIF4E RNAi knockdown or overexpression flies using the gene switching system (RU486), UAS‐eIF4E RNAi and UAS‐eIF4E males were mated with DA‐GSG virgin females on the cornmeal food, respectively. Half of the progeny was cultured on RU486‐containing diets to induce RNAi knockdown or overexpression. The other half was cultured on control diets containing equal amounts of ethanol without RU486. To measure lifespan, adult progeny flies were collected within 24 h after eclosion and mated for another 24 h on the cornmeal and then sorted out by gender. After sorting, flies were placed in vials, each with approximately 20 male or female flies fed the cornmeal for another 24 h. Flies were transferred to vials with different fresh cornmeal once every 2–3 days. The number of dead flies was recorded at the time of transfer. Each lifespan ends when all the flies are dead. For each genotype, lifespan of 100–200 flies in 6–10 vials was measured to determine the mean and maximum lifespan. Maximum lifespan was calculated as the mean lifespan of 10% longest surviving flies.

### 2.4 Automated *C. elegans* Lifespan Analysis

*C. elegans* lifespan analysis was performed using WormBot, as described previously (Pitt et al. 2019). Briefly, animals were maintained on nematode growth medium (wormbook.org) supplemented with 100 units/mL nystatin to prevent fungal growth and a final concentration of 50 μM FUDR to prevent progeny development. For RNA interference (RNAi) experiments, media also contained 100 μg/mL ampicillin, 10 μg/mL tetracycline, and 2 mM IPTG. For all experiments, animals were synchronized by 10‐min hypochlorite treatment (1% sodium hypochlorite, 250 mM potassium hydroxide and water) followed by four washes in M9 buffer to isolate eggs, followed by an overnight hatch‐off in 6 mL M9 in a 60 mm petri dish sealed with parafilm in a 20°C incubator (Torrey Pines Scientific, Torrey Pines, CA). RNAi clones were obtained from the Vidal and Ahringer RNAi feeding libraries (Kamath et al. 2001; Rual et al. 2004), sequence‐verified, and kept as −80°C frozen stocks prior to use. dsRNA induction was performed by growing RNAi feeding strains overnight in media containing antibiotics but without IPTG. The following morning, stationary phase cultures were diluted fourfold with fresh media containing 2 mM IPTG and grown for 4 h at 37°C on a shaker. Following induction, cells were pelleted and resuspended in the original overnight culture volume (4× concentrate) and seeded onto RNAi NGM media. Following 20 μL of seeding with RNAi bacteria, 12‐well plates were allowed to dry for 20–60 min with their lids off in a laminar flow hood to allow complete evaporation of the seeding solution. RNAi experiments were initiated by placing synchronized L1 animals on 10 cm RNAi plates until they reached the L4 larval stage, when they were transferred to the seeded 12‐well RNAi plates containing FUDR. Throughout the experiment, plate humidity was maintained by filling the interstitial spaces of the 12‐well plate with deionized distilled water and refilling as necessary throughout the course of the experiment.

### 2.5 Standard *C. elegans* Lifespan Analysis

To generate the intestine expression vector, a 1.2 kb *vha‐6* promoter containing *Hin*dIII/*Sal*I was cloned into pPD95.75. A *cst‐1* cDNA was subcloned into pPD95.75*Pvha‐6* using *Xba*I/*Kpn*I. DNA sequences were confirmed by Sanger sequencing. Primers used for the DNA constructs are listed in Table S1. pPD95.75*Pvha‐6::cst‐1* (20 ng/μL) was injected into *unc‐119(ed3)* with the co‐injection marker *unc‐119* ^+^. pPD95.75*Pvha‐6* was also injected into *unc‐119(ed3)* to generate control strains.

*C. elegans* strains were maintained at 20°C using standard cultivation techniques, unless otherwise described. *C. elegans* strains were purchased from the Genetic Genome Center or were gifts from other laboratories. Transgenic animals were generated using standard microinjection methods. Mutants were generated using standard genetic methods. All strains were backcrossed at least four times to control strains.

All lifespan analyses were performed at 25°C, unless mentioned otherwise. Synchronized worms were prepared by inducing egg laying for 1 h. Worms were allowed to grow for several days until they reached young adulthood. Synchronized young adult‐stage worms were treated with 0.1 mg/mL FUDR to prevent progeny proliferation. Worms were then scored as dead or alive by tapping with a platinum wire every 1–2 days. Plates were treated with FUDR on day 1 of the lifespan analysis. All lifespan assays were repeated at least three times, unless mentioned otherwise. Statistical analyses and *p* values were calculated using the log‐rank (Mantel‐Cox) method using the OASIS application (http://sbi.postech.ac.kr/oasis2/).

### 2.6 *S. cerevisiae* Replicative Lifespan Analysis

*S. cerevisiae* strains used in the study were obtained from the yeast ORF deletion collection (Giaever and Nislow 2014) and previous studies (Buchan et al. 2008; Yoon et al. 2010). A modified RLS protocol based on previously described methods was utilized (Beaupere et al. 2018; Steffen et al. 2009). Briefly, cells were grown on freshly prepared YPD (2% Yeast Extract, 1% Bacto Peptone, 2% glucose) plates at 30°C until single colonies were visible. Cells were selected from a single colony and lightly patched onto new YPD plates overnight. In the morning, founder cells were aligned and selected as newborn daughter cells using a micromanipulator. Cells were monitored for cell division every 90–120 min, and subsequent budded daughter cells were separated and removed as they formed. The process continued until cell division ceased. Replicative lifespan was calculated as the number of times each mother cell divided before permanent cell cycle arrest. Plates were kept in the refrigerator at 4°C overnight. All experimenters were blinded to the identity of any of the treatments at the time of dissection.

### 2.7 Yeast Growth Analysis

WT BY4741 *S. cerevisiae* and an isogenic *ste20Δ* strain, obtained from the yeast gene knockout library (Winzeler et al. 1999), were grown in synthetic (SD) media (VWR Glucose 2%, Difco yeast nitrogen base 0.17%, Fisher ammonium sulfate 5 g/L, appropriate amino acids, and nucleotides) at 30°C. Overnight cultures were back‐diluted to 0.1 OD_600nm_ in identical media, followed by growth at 30°C to mid‐log (~0.5 OD_600nm_), after which the cultures were adjusted to 0.2 OD_600nm_, serially diluted (1/5 dilutions), and spotted onto 2% agar (Hardy Diagnostics CRITERION) containing SD media.

In biological triplicates, WT BY4741 *S. cerevisiae* transformants were grown overnight in synthetic (SD) media (VWR sucrose 2%, Difco yeast nitrogen base 0.17%, Fisher ammonium sulfate 5 g/L, appropriate amino acids, and nucleotides) at 30°C. Overnight cultures were back diluted to 0.1 OD_600nm_ in identical media, followed by growth at 30°C to mid‐log (~0.5 OD_600nm_), after which the cultures were adjusted to 0.2 OD_600nm_, serially diluted (1/5 dilutions), and spotted onto agar (Hardy Diagnostics CRITERION) plates containing either 0.25% galactose +1.75% sucrose SD media (for modest overexpression) or 2% galactose SD media (for maximal overexpression). Images were captured 2 days after spotting. [IF showing day 7, 14, and 21 data add the following]. Continuous liquid culture of yeast transformants in 2% galactose SD media, followed by equivalent serial dilution analysis at days 7, 14, and 21, was subsequently performed.

### 2.8 Quantification and Statistical Analysis

Data are expressed as means ± SD of at least three independent experiments, as indicated in the corresponding Figure legends. The numbers of biological replicates and what they represent are indicated in each Figure legend. Two‐tailed Student's *t*‐tests were used for single comparisons. *p* < 0.05 was considered statistically significant.

### 2.9 Immunoblot Analysis

*Drosophila* tissues were lysed in 100 μL of protein extraction solution (PRO‐PREP for Cell/Tissue, iNtRON BIOTECHNOLOGY) and dissolved in 4× Laemmli sample buffer. Precision Plus Protein Dual Color Standards (BIO‐RAD) were used as a marker. The membrane was washed three times for 10 min with TBST (900 mL distilled water, 100 mL 10× TBS [#HTBS‐1010, HanLAB], 1 mL Tween 20[#1706531, BIO‐RAD]). Blocking was processed in 5% skim milk (Difco Skim Milk, #232100, Becton, Dickinson and Company) in TBST for 1 h. Primary antibodies in 5% skim milk were incubated overnight at 4°C, and secondary antibodies in 5% skim milk were incubated for 2 h at room temperature. The membranes were washed 6 times for 10 min with TBST and treated with SuperSignal West Pico PLUS Chemiluminescent Substrate (Thermo Scientific, #34578). The membrane detection and quantification were obtained with Alliance Q9 (UNITEC Cambridge).

Whole‐cell lysates were prepared in radioimmunoprecipitation assay (RIPA) buffer, separated by sodium dodecyl sulfate‐polyacrylamide gel electrophoresis (SDS‐PAGE), and transferred onto nitrocellulose membranes (Invitrogen iBlot Stack). Immunoblots were performed using antibodies targeting Tuba1a (1:5000, SCBT sc‐5286), MST1 (1:1000, CST #3682), p‐MST1 (1:500, CST #49332), AUF1 (1:2000, Millipore 07–260), HuR (1:2000, SCBT sc‐5261), QKI (1:1000, Abcam ab126742), HA (1:5000, Sigma 11867423001), hnRNP K (1:1000, SCBT sc‐28380), EDC3 (1:1000, SCBT sc‐365024), eIF4E (1:1000, SCBT sc‐9976), Rck (1:1000, SCBT sc‐376433), Dcp2 (1:1000, Abcam ab28658), NCL (1:1000, Abcam ab13541), Akh (*Drosophila*, 1:200, generated by Dr. KS Lee), Act5c (*Drosophila*, 1:3000, Developmental Studies Hybridoma Bank, University of Iowa, JLA20), Actb (1:5000, SCBT sc‐47778), acetyl‐CoA carboxylase 1 (ACC1) (1:1000, CST #4190), fatty acid‐binding protein 4 (FABP4) (1:1000, CST #13368), and fatty acid synthase (FASN) (1:1000, SCBT sc‐55580). Horseradish Peroxidase (HRP)‐conjugated secondary antibodies were purchased from GE Healthcare. The antibody against *Drosophila* Pxn was a gift from Dr. Jiwon Shim, and the Akh antibody was generated by K.‐S.L. in KRIBB.

## 3 RNA‐Sequencing Analysis

To perform RNA‐seq experiments, total RNA was extracted from 50 larval fat body tissues dissected and isolated using the easy‐BLUE reagent (iNtRON Biotechnology), following the manufacturer's protocol. The experimental groups included *Dcg/+* (control), *Dcg* > *Hpo* (Hpo overexpression), and *Dcg* > *Hpo RNAi* (Hpo knockdown), where *Hpo* was manipulated in a fat body‐specific manner. RNA quality was assessed to ensure high integrity and purity before sequencing, and RNA‐seq library preparation and data analyses were conducted by ebiogen (ebiogen.com). The RNA‐seq data have been deposited in the NCBI GEO database under the accession code GSE201903.

## 4 Results

### 4.1 Hpo Overexpression Decreases Lifespan and Lipid Metabolism in *Drosophila melanogaster*

**FIGURE 1:**
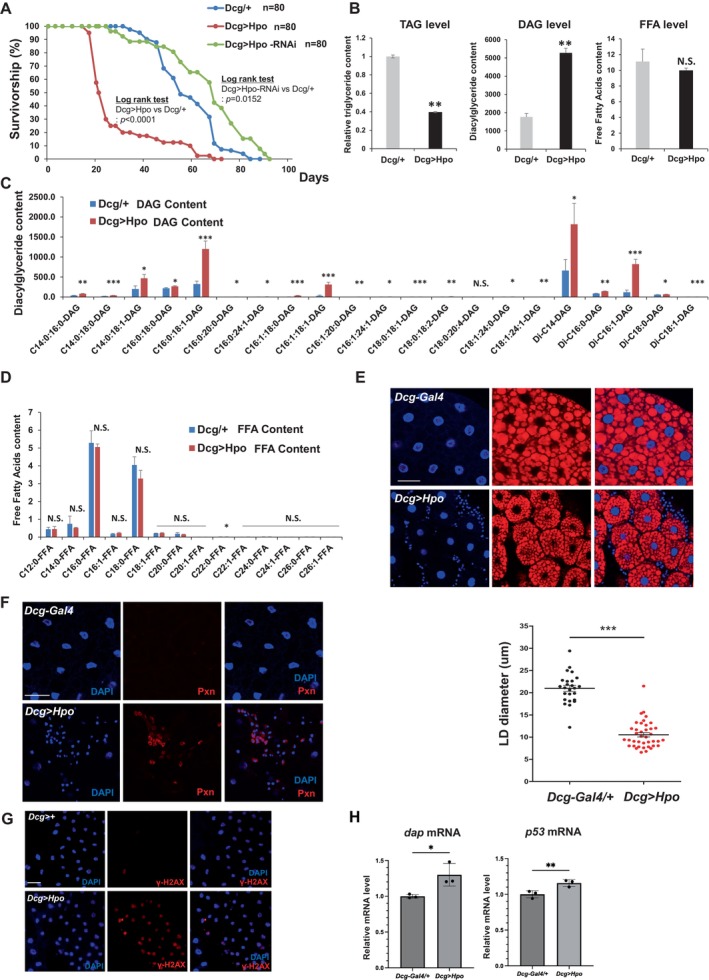
Hippo overexpression decrease of lifespan and promotion of lipolysis in *Drosophila*. (A) Survivorship analysis of wild‐type (Dcg/+), Hippo‐overexpressing (Dcg > Hpo), and Hippo RNAi (Dcg > Hpo‐RNAi) *D. melanogaster* lines from newly eclosed male and female flies. *N* = 80. (B) Levels of TAG, DAG, and FFA species measured by TAG assay and HPLC‐MS/MS analyses of third instar larval homogenates isolated from Dcg/+ and Dcg > Hpo lines. Error bars represent SD of three independent experiments. *N* = 3; **p < 0.01; NS, not significant from Student's *t*‐test. (C, D) Level of DAG (C) and FFA (D) species measured by HPLC‐MS/MS of larval homogenates isolated from Dcg/+ and Dcg > Hpo lines. Error bars represent the SD of three independent experiments. *N* = 3; ***p < 0.001; **p < 0.01; *p < 0.05; NS, not significant from Student's *t*‐test. (E) Staining of fat bodies of third instar larvae from Dcg/+ and Dcg > Hpo lines using nile red (red) and DAPI labeling of nuclei (blue). Quantification of lipid droplet (LD) diameter in Dcg/+ and Dcg > Hpo animals. Each point represents a single LD. Error bars represent SD. *N* = 3, ***p < 0.001 from Student's *t*‐test. Scale bar, 50 μm. (F) Staining of fat bodies of third instar larvae from Dcg/+ and Dcg > Hpo animals using anti‐Pxn antibody as a marker of infiltrating immune cells. Scale bar, 50 μm. (G) Staining of fat bodies of third instar larvae from Dcg/+ and Dcg > Hpo animals using anti‐gamma‐H2AX antibody as a marker of senescence. Scale bar, 50 μm. (H) mRNA levels of *dap* and *p53* from RT‐qPCR data of Dcg > Hpo. Error bars represent the SD of three independent experiments. *N* = 3, **p < 0.01, *p < 0.05 from Student's *t*‐test.

To evaluate the role of Hippo in aging, we first examined the lifespan of *Drosophila melanogaster* selectively overexpressing hpo in the fat body (*Dcg‐Gal4*). Survivorship analysis revealed that hpo overexpression shortened median survival from 54 to 21 days (*p* < 0.0001, 80 total female and male *Drosophila*) and maximum lifespan (Figure 1A). Measurement of *hpo* mRNA by RT‐qPCR (primers listed in Table S1) revealed a 39‐fold increase in *hpo* mRNA in the fat bodies of flies overexpressing hpo (Figure S1A). Contrastingly, knockdown of Hpo by RNAi extended median survival from 54 to 68 days (*p* = 0.0152) (Figure 1A). Maximum survival was not significantly affected.

We also evaluated the effect of whole‐body Hpo overexpression and deletion on lifespan in *Drosophila melanogaster* during the adult stage (*Daughterless‐GeneSwitch‐Gal4*, *Da‐GSG*) using treatment with the progesterone antagonist mifepristone/RU486 (Osterwalder et al. 2001) (Figure S1B). Survivorship analysis revealed that ubiquitous Hpo overexpression shortened median survival from 88 to 70 days (*p* < 0.0001 from 152 to 155 total female *Drosophila*, respectively) and decreased maximum lifespan (99 days in untreated animals and 92 days RU486‐treated animals) (Figure S1B). Whole‐body depletion of Hpo using RNAi marginally decreased lifespan (median lifespan 77 days for untreated animals and 69 days for RU486‐treated animals; maximum lifespan 95 days for untreated animals and 92 days for RU486‐treated animals), which differed from fat body‐specific RNAi (*p* < 0.0001 in 172 and 164 female *Drosophila*, respectively) (Figure S1B). The results of these inducible experiments are consistent with those observed in fat body‐specific lines, supporting the physiological relevance of our findings. These data demonstrate that inducible, adult‐specific overexpression and knockdown of Hpo recapitulate the phenotypes observed in tissue‐specific lines, while providing additional evidence that the observed effects are not solely attributable to developmental overexpression or toxicity. In whole‐body overexpression lines, *hpo* mRNA was upregulated 38.9‐fold (*n* = 5 female heads) and depleted ~50% in RNAi lines (Figure S1A). Transcripts encoding proteins involved in lifespan regulation, the immune response, and lipolysis were also altered in Dcg > Hpo lines (Figure S1C). These results were consistent with the notion that Hpo contributes significantly to longevity in *Drosophila*.

Because our results differed from those previously reported in *C. elegans* (Lehtinen et al. 2006), we independently examined the effect of overexpressing the *C. elegans* Hpo homolog cst‐1. As previously observed, cst‐1 overexpression under a ubiquitous promoter slightly increased lifespan (Figure S2A, left). Since the intestine is the major fat storage tissue in *C. elegans* (Musselman and Kühnlein 2018), we generated intestine‐specific cst‐1 expression (isoform A or D) using a V‐ATPase a‐subunit vha‐6 promoter and observed that cst‐1 overexpression marginally increased lifespan compared to control animals (Figure S2A, right). Deletion of the *S. cerevisiae* Hpo homolog sterile 20 (ste20) did not affect *S. cerevisiae* growth rates in the stationary phase relative to the wild‐type strain (Figure S2C). However, Ste20 overexpression using galactose media decreased yeast survival during the stationary phase (Figure S2B). Taken together, these findings revealed that Hpo dosage regulated lifespan in *D. melanogaster* , *C. elegans* , and *S. cerevisiae* , but that the directionality was different and could depend on tissue‐specific expression levels.

### 4.2 Hpo Overexpression Promotes Lipolysis in the *Drosophila* Fat Body

Because fat body‐specific Hpo overexpression shortened lifespan, we hypothesized that fatty acid metabolism was also affected in Hpo‐overexpressing animals. To test this hypothesis, we isolated fat body tissue from wild‐type and Hpo‐overexpressing animals and quantified fatty acid abundance. Total tri‐acylglyceride (TAG) levels were decreased, di‐acyl glyceride (DAG) was significantly increased, while free fatty acids (FFAs) were not changed (Figure 1B). Profiling of individual lipids revealed that almost all species of DAGs, but not of FFAs, were highly upregulated in Hpo‐overexpressing lines (Figure 1C,D). The major lipolytic enzymes adipose triglyceride lipase (ATGL), hormone‐sensitive lipase (HSL), and monoglyceride lipase (MGL) were upregulated in Hpo‐overexpressing animals (Figure S1D).

Nile red staining revealed that Hpo overexpression decreased lipid droplet sizes in larval fat bodies (Figure 1E). We also identified that many small nuclei were present in DNA staining around fat bodies overexpressing Hpo compared to wild‐type (Figure 1E,F). These consisted primarily of immune cells, as evidenced by staining with a macrophage‐like plasmatocyte‐specific antibody, anti‐Pxn antibody (Figure 1F). Infiltration of immune cells, including macrophages, into adipose tissue is a prominent feature of adipose tissue dysfunction in mammals (Exley et al. 2014; Kang and Lee 2024; Chavakis et al. 2023). These findings demonstrated that fat body‐specific Hpo overexpression resulted in functional adipose tissue defects, including decreased lipid droplet size and TAG, abnormal DAG accumulation, and immune cell infiltration. To check whether Hpo overexpression also affects senescence and cancer risk, we also examined Histone γ‐H2AX as a potential marker of cellular senescence. We observed that γ‐H2AX foci are increased in the Hpo overexpression background (Figure 1G). Also, RT‐qPCR data show elevated levels of senescence‐associated mRNAs, including *dap* and *p53* (Figure 1H).

### 4.3 Hpo Overexpression Increased Expression of Lipogenic mRNAs and RNA‐Binding Protein mRNAs

To identify the molecular mechanisms for Hpo suppression of fatty acid metabolism and/or promotion of lipid anabolism, we performed high‐throughput RNA‐seq analysis using total RNA purified from wild‐type control (Dcg‐gal4/+), Hpo‐overexpressing (Dcg > Hpo), and Hpo knockdown (Dcg > Hpo‐RNAi) animals. *Hpo* mRNA was significantly increased with Hpo overexpression (33.3‐fold upregulation) and decreased by Hpo knockdown (0.552‐fold downregulation) relative to control (Dcg‐gal4/+). In Dcg > Hpo animals, 244 transcripts were upregulated, and 4064 transcripts were downregulated (> 2‐fold) relative to wild‐type animals (Table S2). In Hpo knockdown animals, 1382 transcripts were upregulated, and 2256 transcripts were downregulated (> 2‐fold) relative to control (Table S2). Gene Ontology analysis enriched G protein‐coupled receptor signaling pathways (FDR: 7.31E^−15^), hormone activity (FDR: 8.45E^−08^), protein localization to mitochondria (FDR: 6.83E^−06^), and nucleosome assembly (FDR: 7.72E^−05^) (Table S3). To determine whether Hpo overexpression and knockdown affect the abundance of transcripts in opposite directions, we analyzed genes associated with lipid metabolism‐related GO terms (Figure S1E). To provide a clearer illustration of the regulation of these genes, we included a four‐way Venn diagram with the list of the overlapping genes that are upregulated in Hpo overexpression and downregulated in Hpo knockdown (Figure S1F). In addition, we extended our analysis to genes associated with aging and immune response GO terms. Our results confirmed that a subset of genes within these categories exhibit opposite directions of changes between Hpo overexpression and knockdown conditions (Figure S1G).

**FIGURE 2:**
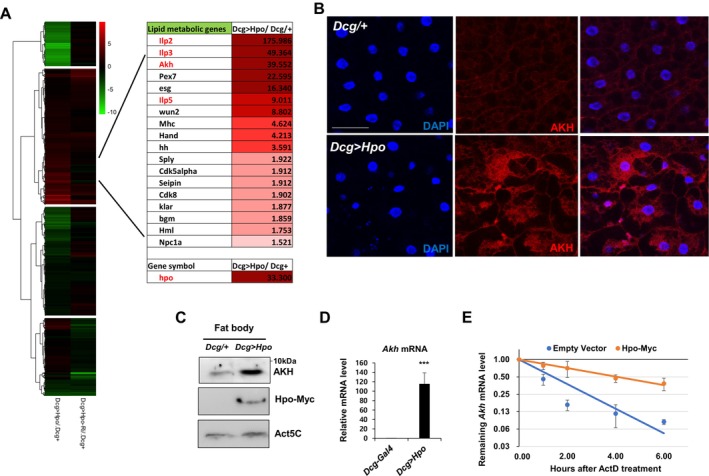
Hippo overexpression upregulation of lipid metabolism and RNA‐binding protein genes in *Drosophila*. (A) Heatmap of RNA‐seq using total RNA isolated from fat bodies of third instar larvae of Dcg/+ and Dcg > Hpo animals. Relative abundance of transcripts encoding lipid metabolism genes (*top*) and hpo were plotted in Dcg > Hpo animals. (B) Immunostaining of fat bodies of third instar larvae isolated from Dcg/+ and Dcg > Hpo animals with Akh (red) and DAPI (blue). Scale bar, 50 μm. (C–E) Protein and mRNA levels were measured by immunoblot (Akh, Myc, and Act5C) and RT‐qPCR (*Akh* mRNA) in fat bodies of third instar larvae of Dcg/+ and Dcg > Hpo animals. *Akh* mRNA stability was measured after transcription inhibition using actinomycin D in Dcg/+ and Dcg > Hpo animals. *N* = 3, ***p < 0.001 from Student's *t*‐test.

Among transcripts inversely fluctuating in Dcg > Hpo relative to Dcg > Hpo‐RNAi animals, we further evaluated transcripts encoding proteins involved in lipid metabolism, identifying that mRNA levels of insulin‐like peptide 2 (Ilp2), Ilp3, Ilp5, and Akh were highly increased in Dcg > Hpo animals (Figure 2A). Immunostaining and Western blot analysis with the Akh antibody confirmed that Akh was highly overexpressed in fat bodies isolated from Dcg > Hpo animals (Figure 2B,C). The increased protein level of Hpo was also shown by Western blot analysis in Hpo‐overexpressing flies (Figure 2C). We demonstrated that the accumulation of *Akh* mRNA (Figure 2D) was primarily due to increased stability using transcription shut‐off and RT‐qPCR analysis of S2 cells transfected with empty or Hpo‐Myc plasmids (Figure 2E). These results demonstrated that Hpo stabilized transcripts involved in fatty acid metabolism and their post‐transcriptional regulators, promoting the rapid breakdown of TAG to DAG.

**FIGURE 3:**
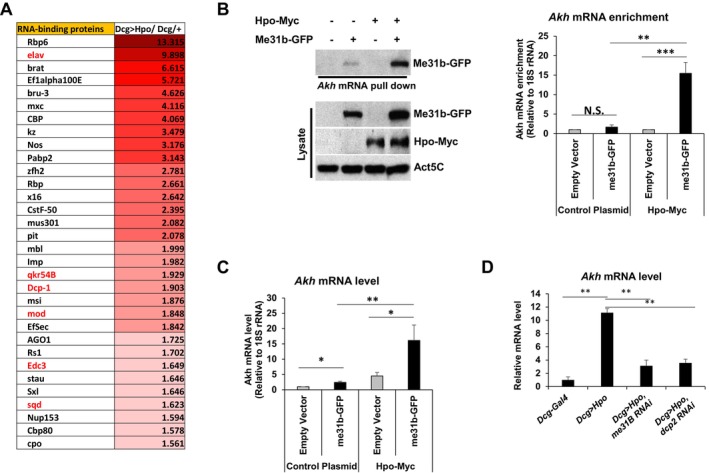
Hippo overexpression upregulates Akh expression level. (A) Relative abundances of transcripts encoding RNA‐binding proteins (*top*) are plotted according to their degrees of fold changes. (B) *Akh* mRNA pull‐down (*left*) and Western blot of Me31b‐GFP (*right*) using S2 cell lysates. *Akh* mRNA RT‐qPCR of me31b‐GFP immunopellets from lysates of S2 cells transfected with empty vector or a Hpo‐Myc‐expressing plasmid. *N* = 3, ***p < 0.001, **p < 0.01, *p < 0.05, NS, not significant from Student's *t*‐test. (C) *Akh* mRNA level in S2 cells transfected with plasmids expressing me31b‐GFP or Hpo‐Myc relative to empty vector control. *N* = 3, **p < 0.01, *p < 0.05 from Student's *t*‐test. (D) *Akh* mRNA level in fat bodies of third instar larvae of Dcg/+, Dcg > Hpo, Dcg > Hpo/Me31b RNAi, and Dcg > Hpo/Dcp2 RNAi animals. *N* = 3, **p < 0.01 from Student's *t*‐test.

To identify RNA‐binding proteins that regulated *Akh* mRNA stability, we profiled proteins interacting with *Akh* mRNA in S2 cell lysates (Ko et al. 2021). Anti‐sense oligo (ASO)‐mediated pull‐down of *Akh* mRNA and mass spectrometry of co‐purified proteins revealed that *Akh* mRNA interacted with the RNA‐binding proteins La‐related protein (LARP), LARK, and Modulo (mod) (Ko et al. 2021). Reflecting the data of the *Akh* mRNA interactome by using ASO pull‐down and Hpo overexpression‐dependent transcriptome, we identified several RBPs as shown on the heatmap (Figure 3A). Among these proteins, transcripts encoding mod were overexpressed in Dcg > Hpo animals. Similarly, embryonic lethal abnormal vision (Elav), quaking (Qki), Mod, Edc3, Squid (Sqd), and *Drosophila* fragile X mental retardation 1 (dFMR1) mRNA levels were upregulated in Dcg > Hpo animals (Figure 3A). Given that the level of *Me31b* mRNA was elevated in Dcg > Hpo, several key RBPs are enriched in the *Akh* mRNA pull‐down (Ko et al. 2021), and Me31b functions in maternal mRNA decay in *Drosophila* (Wang et al. 2017), we prioritized Me31b as a potential regulator of *Akh* mRNA. RNA affinity pull‐down of *Akh* mRNA enriched Me31b in *Drosophila* S2 cell lysates (Figure 3B, *left*), while Hpo overexpression promoted the interaction of *Akh* mRNA with Me31b (Figure 3B, *right*). Immunoprecipitation of GFP‐tagged Me31b also enriched *Akh* mRNA, which was enhanced by Hpo overexpression (Figure 3C). Importantly, *Akh* mRNA was even further increased when Me31b and Hpo were co‐expressed. Further, increased *Akh* mRNA and protein levels in Dcg > Hpo animals were reversed when the Dcg > Hpo line was crossed with either Me31b or Dcp2 RNAi lines (Figure 3D). These results suggested that *Akh* mRNA was stabilized by Hpo‐mediated overexpression of RNA‐binding proteins, potentially by direct phosphorylation.

### 4.4 Mouse Mst1 Promoted Adipocyte Differentiation and Lipolysis

**FIGURE 4:**
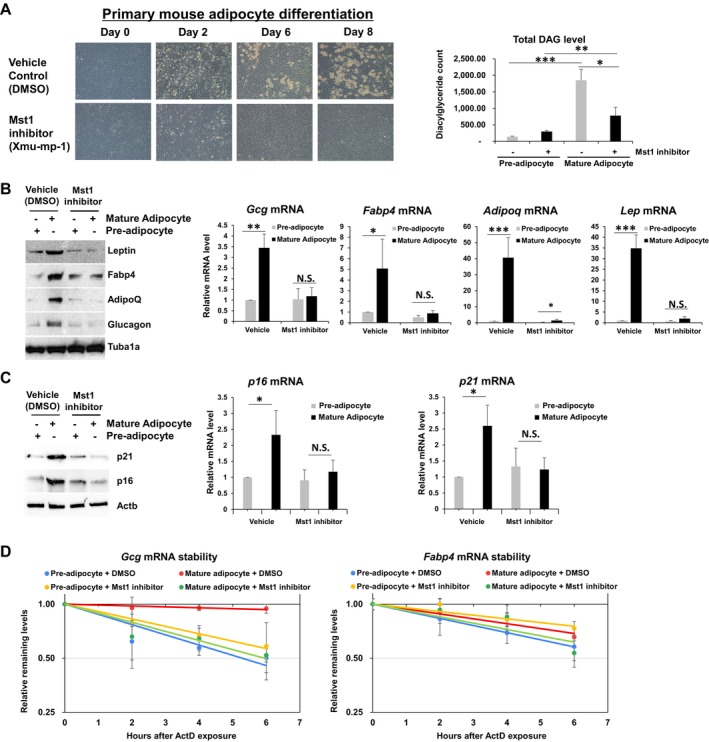
Hippo promotion of mouse adipocyte differentiation via RNA‐binding protein phosphorylation. (A) Phase contrast images (*left*) and total DAG levels (*right*) of primary mouse adipocytes on days 0, 2, 6, and 8 after differentiation and co‐treatment with the Mst1 inhibitor Xmu‐mp‐1 (5 μM) or vehicle (DMSO) beginning on day 0. *N* = 3, ***p < 0.001, from Student's *t*‐test. (B) mRNA (RT‐qPCR, *right*) and protein (Western blot, *left*) levels of *Lep*, *Fabp4*, *Adipoq*, and *Gcg* mRNAs and proteins from pre‐adipocytes (Day 0) and mature adipocytes (Day 8) after treatment with the Mst1 inhibitor or vehicle. Values are expressed as mean ± SD of three independent experiments (*p* < 0.001, Student's *t*‐test). *N* = 3, ***p < 0.001, **p < 0.01, *p < 0.05, NS, not significant from Student's *t*‐test. (C) mRNA (RT‐qPCR, *right*) and protein (Western blot, *left*) levels of *p16*, *p21*, and *Actb* mRNAs and proteins from pre‐adipocytes (Day 0) and mature adipocytes (Day 8) after treatment with the Mst1 inhibitor or vehicle. Values are expressed as mean ± SD of three independent experiments (*p* < 0.001, Student's *t*‐test). *N* = 3, *p < 0.05, NS, not significant from Student's *t*‐test. (D) *Gcg* and *Fabp4* mRNA stabilities were measured after inhibiting transcription with Actinomycin D in mouse 3 T3‐L1 cells before and after differentiation and with or without treatment with the Mst1 inhibitor. Data were normalized to 18S rRNA.

To determine if the role of Hpo in adipogenesis and lipid metabolism was conserved in mammals, we studied the role of the mammalian Hippo homolog Mst1 in an in vitro model of primary mouse adipocyte differentiation. Administration of the Mst1 inhibitor Xmu‐mp‐1 in primary adipocytes delayed differentiation in a dose‐dependent manner (Figure 4A *left* and Figure S3). We introduced Mst1 shRNAs by using the lentiviral constructs into primary adipocytes or 3T3‐L1 cell lines, and then observed changes similar to Xmu‐mp‐1 treatment (Figure S2D–F). DAG profiles revealed that increased DAG production in mature adipocytes was suppressed by treatment with the Mst1 inhibitor (Figure 4A *right* and Figure S3A). Delayed adipocyte differentiation was confirmed by western blot and RT‐qPCR analyses of adipogenic proteins (Figure 4B, *left*). mRNAs such as leptin (*Lep*), fatty acid binding protein 4 (*Fabp4*), adiponectin (*Adipoq*), and glucagon (*Gcg*), which are normally induced 8 days after differentiation, were not induced after administration of the Mst1 inhibitor (Figure 4B, *right* and Figure S3B). Treatment with the ROS scavenger N‐acetyl‐L‐cysteine or the cytochrome P450 family 2 subfamily E member 1 (CYP2E1) inhibitor chloromethiazole (Cui et al. 2019; Eap et al. 1998; Hu et al. 1994) did not affect adipocyte differentiation (Figure S3C–D). These results demonstrated that Mst1 was essential for mouse adipocyte differentiation independent of ROS generation. Furthermore, we have examined the levels of *p16* and *p21* mRNAs as senescence markers, and then identified that Xmu‐mp‐1 treatments suppressed induction of *p16* and *p21* mRNAs upon 3 T3‐L1 differentiation as suggested in Fan et al. (2020) Aging (Figure 4C). We found that Mst1 depletion by shRNAs in primary adipocytes does not promote senescence and rather its depletion suppresses induction of *p16* and *p21* mRNA as a marker of senescence (Figure S4C).

### 4.5 Mouse Mst1 Phosphorylated RNA‐Binding Proteins to Stabilize *Glucagon* mRNA for Adipocyte Differentiation

These findings prompted the hypothesis that Mst1 promotes adipocyte differentiation by stabilizing *Glucagon*, a mammalian orthologue of *Drosophila Akh*, via Mst1‐mediated phosphorylation of RNA‐binding proteins. Our hypothesis was supported in part by the finding that *Gcg* mRNA, but not *Fabp4 mRNA*, was stabilized during adipocyte differentiation in an Mst1‐dependent manner (Figure 4D). The half‐life of *Gcg* mRNA in precursor adipocytes is 5.1 h but increases to 42.4 h in mature adipocytes. Treatment with the Mst1 inhibitor decreased *Gcg* mRNA half‐life to 5.6 h 8 days after initiating the differentiation process. These results revealed that Mst1 contributes to the stabilization of *Gcg* mRNA during adipocyte differentiation.

**FIGURE 5:**
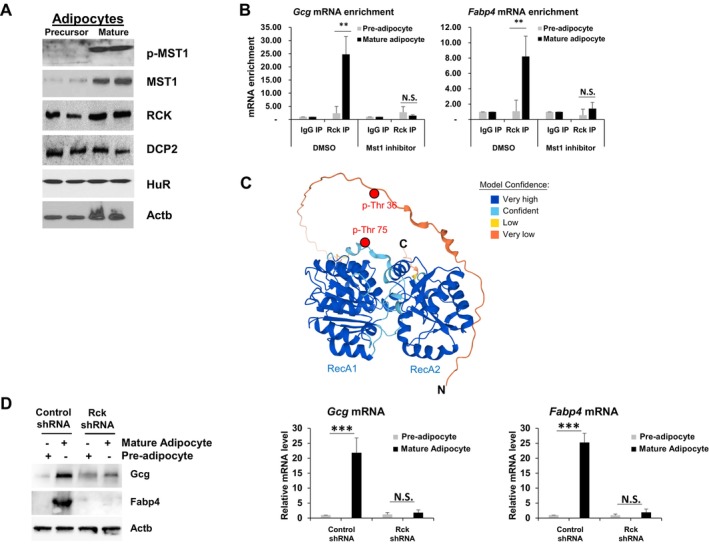
Prediction of human RCK structure. (A) Western blot analysis of p‐MST1, MST1, HuR, Rck, DCP2, and Actb in cell lysates derived from pre‐adipocytes (Day 0) and mature adipocytes (Day 8) from Student's *t*‐test. (B) RT‐qPCR levels of *Fabp4* and *Gcg* mRNAs normalized to *Gapdh* mRNA purified from Rck or IgG immunopellets from cell lysates of precursor and mature adipocytes. Data are expressed as mean ± SD of three independent experiments. *N* = 3, **p < 0.01, NS, not significant from Student's *t*‐test. (C) Human RCK structure predicted using the AlphaFold database provided by Deepmind and EMBL. The colors indicate the accuracy of the model based on a per‐residue confidence score (pLDDT) between 0 and 100. Blue represents pLDDT values over 90 (Very high). Sky blue indicates pLDDT values between 70 and 90 (Confident). Yellow color represents pLDDT values between 50 and 60 (Low). Deep orange color indicates the pLDDT values below 50 (Very low). The phosphorylation sites, Thr 36 and Thr 75, are indicated by the red circles based on the direction of the side chain. (D) mRNA (RT‐qPCR, *right*) and protein (Western blot, *left*) levels of *Fabp4*, *Gcg*, and *Actb* mRNAs and proteins from pre‐adipocytes (Day 0) and mature adipocytes (Day 8) after transfection of the Rck shRNA or control. Values are expressed as mean ± SD of three independent experiments (*p* < 0.001, Student's *t*‐test). *N* = 3, ***p < 0.001, NS, not significant from Student's *t*‐test.

Our previous studies in *S. cerevisiae* demonstrated that *Ste20* mediates phosphorylation of decapping enzyme 2 (Dcp2) in response to oxidative stress (Yoon et al. 2010). We also demonstrated that the human *Ste20* homolog, MST1, phosphorylates eukaryotic mRNA translation initiation factor 4E (eIF4E) (Min et al. 2017), AUF1 (Mun et al. 2024), QKI, and NCL (Chung et al. 2024). To determine if MST1 couples adipocyte differentiation and *Glucagon* mRNA stabilization via its protein kinase activity, we examined MST1 activation, as measured by MST1 auto‐phosphorylation at T183 (p‐MST1) in whole‐cell lysates prepared from precursor and mature adipocytes. MST1 and p‐MST1 (Thr‐183) levels were significantly elevated in mature adipocytes, and RCK, DCP2, and human antigen R (HuR) expressions were unaltered in mature adipocytes relative to precursor adipocytes (Figure 5A). Our findings suggest the involvement of MST1 in signaling pathways that regulate Glucagon protein levels by tightly regulating the stability of its transcript.

MST1 is a Ser/Thr kinase, and we previously demonstrated that MST1 phosphorylates DCP2 and eIF4E. Moreover, our findings suggest that MST1 modulates *Glucagon* mRNA levels by stabilizing its transcript. The RNA‐binding protein RCK, a known regulator of RNA stability (Hu et al. 2015), binds *Gcg* and *Fabp4* mRNA (Figure 5B), similar to our observations of *Akh* mRNA in *Drosophila* S2. Because the interaction of RCK with *Gcg* and *Fabp4* mRNA is abolished by treatment with an Mst1 inhibitor (Figure 5B), we postulated that Rck could be an MST1 substrate and that RCK phosphorylation could affect *Gcg* and *Fabp4* mRNA stabilities. To test this hypothesis, we used recombinant MST1, Rck, and EDC3 proteins to perform in vitro phosphorylation reactions with Mst1 and the RNA‐binding proteins (Figure S3E). MST1 phosphorylated Thr‐36 and Thr‐75 residues in recombinant RCK protein (Figure S3F). We have tested the effect of Rck knockdown in primary adipocytes and confirmed the similar effects on adipocyte differentiation (Figure 5D). The RCK structure, predicted using the AlphaFold database provided by Deepmind and EMBL, was used to predict the MST1 phosphorylation site (Figure 5C). Taken together, these results suggest that MST1 directly phosphorylated Rck, a novel substrate, to regulate *Gcg* mRNA stability during adipocyte differentiation.

### 4.6 Critical Role of Hpo/Mst1‐Mediated RNA‐Binding Protein Phosphorylation in Regulation of Adipogenic Aging

**FIGURE 6:**
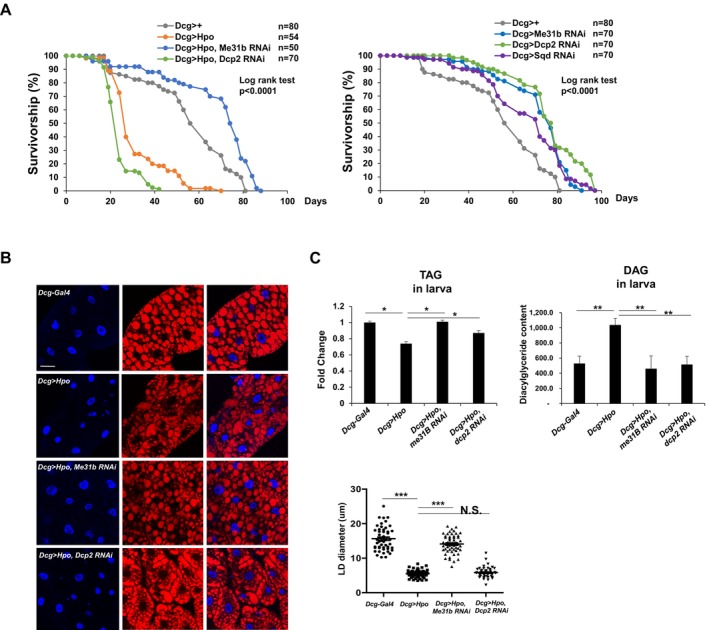
Requirement of Me31b/RCK for Hippo‐mediated decrease of lifespan. (A) Survivorship analysis of wild‐type (Dcg > +) and Hippo‐overexpressing (Dcg > Hpo) animals crossed with Me31b RNAi (Dcg > Hpo/Me31b‐RNAi), and Dcp2 RNAi (Dcg > Hpo/Dcp2‐RNAi) *D. melanogaster* . *N* = 80 (Dcg > +), 54 (Dcg > Hpo), 50 (Dcg > Hpo, Me31b RNAi) and 70 (Dcg > Hpo, Dcp2 RNAi; Dcg > Me31b RNAi; Dcg > Dcp2 RNAi and Dcg > Sqd RNAi). (B) Lipid droplet staining of fat bodies of third instar larvae from *Dcg/+*, *Dcg* > *Hpo*, *Dcg* > *Hpo*/Me31b RNAi and *Dcg* > *Hpo*/Dcp2 RNAi animals using nile red and DAPI labeling of nuclei (blue). Scale bar, 50 μm. Quantification of LD diameter, with each point representing a single LD. Data are expressed as mean ± SD. *N* = 3; ***p < 0.001; NS, not significant from Student's *t*‐test. (C) Tri‐acyl glycerol (TAG) levels in homogenates purified from third instar larvae of Dcg/+, Dcg > Hpo, Dcg > Hpo/Me31b RNAi and *Dcg* > *Hpo*/Dcp2 RNAi animals. Data are expressed as mean ± SD of three independent experiments. *N* = 3, **p < 0.01, *p < 0.05 from Student's *t*‐test.

Since we observed that Hpo overexpression results in a shorter lifespan in *Drosophila*, a reduction of fatty acid metabolism, and stabilization of adipogenic or lipogenic mRNAs, we reasoned that Hpo targets specific RNA‐binding proteins to modulate adipogenic aging. To test this model in vivo, we crossed the DCG > Hpo *Drosophila* line with RNAi lines targeting RNA‐binding proteins phosphorylated by Hpo and its homologs in other metazoans (Min et al. 2017; Yoon et al. 2010). These proteins included eIF4E, Me31b/RCK, Dcp2, Sqd/Heterogeneous Nuclear Ribonucleoprotein D (hnRNPD), NCL, and Edc3. Survivorship analysis revealed that Hpo‐mediated reduction of *Drosophila* lifespan was partially reversed in *Dcg* > *Hpo/Me31b RNAi* animals, but not in *Dcg* > *Hpo/Dcp2 RNAi* animals (Figure 6A *left*). Depletion of individual RNA‐binding proteins using RNAi in *Drosophila* demonstrated that RNAi or overexpression of eIF4E caused no significant changes to *eIF4E* mRNA levels (Figure S4A,B), but the median lifespan of eIF4E RNAi animals was slightly decreased. On the other hand, individual depletion of Dcp2, Sqd, and Me31b improved lifespan (Figure 6A *right*). Taken together, these findings demonstrate that Me31b, the *Drosophila* orthologue of RCK, is a major target of RNA‐binding proteins essential for Hpo lifespan regulation in *Drosophila*.

We also examined the lifespan of *C. elegans* and *S. cerevisiae* lines lacking cst‐1 and Ste20, the RNA‐binding proteins targeted by Hpo homologs. Depletion of eIF4E or CGH‐1/RCK in *C. elegans* did not alter lifespan (Figure S5A). Deletion of Dcp1 or Pat1 in *S. cerevisiae* dramatically reduced the replicative lifespan, primarily due to increased cell death (Figure S5B). Deletion of Edc3 in *S. cerevisiae* increased lifespan, and deletion of the Lsm4 C‐terminal together with Edc3 abolished this phenotype (Figure S5C). Changes in the replicative lifespan in *S. cerevisiae* were reflected by changes in gene expression analyzed by cDNA microarray analysis of these strains. Deletion of Edc3 primarily decreased levels of mRNAs encoding mitochondrial proteins (Figure S5D), and these changes were reversed by deletion mutations in the Sm‐like proteins 4 (Lsm4) carboxyl‐(C‐) terminal domain (Figure S5E) or Dcp1 (Figure S5F). Genetic deletion of Pat1 or Dhh1/RCK upregulated mRNAs encoding ribosomal proteins (Figure S5G). These findings suggest differential functions of the mRNA decay proteins targeted by Hpo and its homologs in metazoans.

**FIGURE 7:**
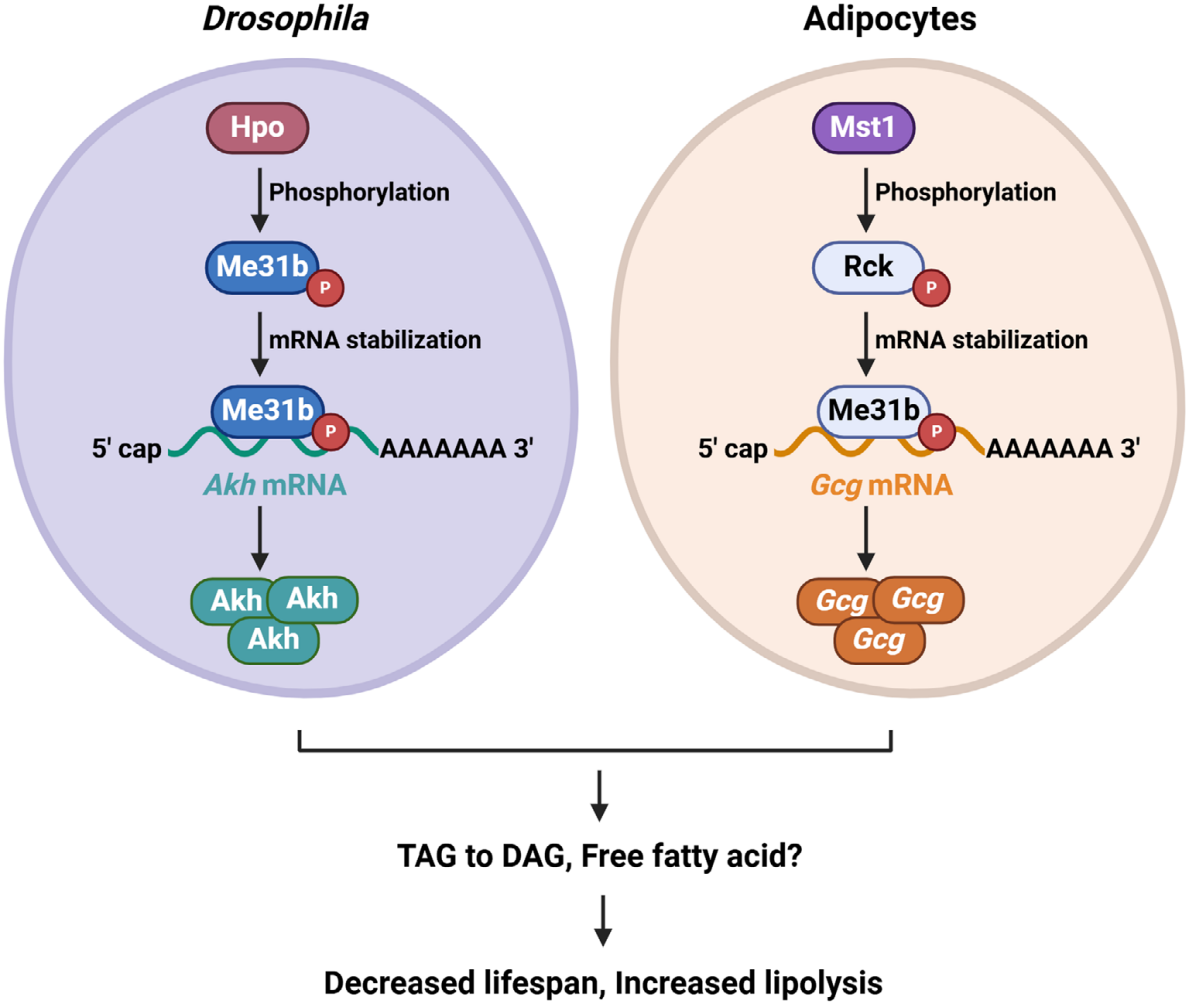
Proposed model for Hpo regulation of lifespan. Proposed model for Hpo regulation of lifespan.

Finally, we examined lipid levels and lipid droplet sizes of *Drosophila* fat bodies in these lines (Figure 6B,C). Nile red staining of lipid droplets revealed that Hpo overexpression decreased lipid droplet (LD) sizes in larval fat bodies, which was partially reversed by co‐expression of Me31b RNAi (Figure 6B). However, lipolysis resulting from Hpo overexpression was not rescued by Dcp2 RNAi (Figure 6B). Decreased TAG in *Dcg* > *Hpo* animals was most effectively restored in *Dcg* > *Hpo/Me31b RNAi* animals (Figure 6C and Figure S6A) while there were no significant changes in TAG levels in Me31b, Dcp2, or Sqd RNAi lines without Hpo overexpression (Figure S6B). Taken together, these findings demonstrate that the Hpo pathway contributes to the regulation of *Drosophila* lifespan by modulating the expression and activity of Me31b/RCK, which subsequently affects mRNA levels of adipogenic or lipogenic genes (Figure 7).

## 5 Discussion

In the present study, we identified that *Drosophila* Hpo regulates animal lifespan and immune infiltration of adipose tissue. Our findings are supported by observations that Hpo overexpression (1) decreased median and maximum lifespan, (2) increased the stability of mRNA encoding *Akh*, a systemic lipogenic hormone, (3) the mouse Hpo homolog Mst1 was essential for adipocyte differentiation and *Gcg* mRNA stabilization and (4) the RNA‐binding protein Me31b/Rck was critical for Hpo/Mst1‐mediated *Akh*/*Gcg* mRNA stabilization. Marginal changes in lifespan in Hpo‐depleted *Drosophila* lines also suggest the potential roles of additional target proteins in modulating *Drosophila* lifespan, lipid metabolism, and tissue specificity (Figure 1A).

### 5.1 Regulatory Roles of Hippo Pathway and RNA‐Binding Proteins in *Drosophila* Lifespan

The Hippo pathway is known to regulate lifespan in *C. elegans* and to regulate the activity of DAF‐16/FOXO (Lehtinen et al. 2006). In *C. elegans* , cst‐1 depletion decreases lifespan, fast body movement, and pharyngeal pumping rate. Whole‐body or intestine‐specific cst‐1 overexpression increases lifespan, while additional DAF‐16 mutations suppress lifespan. However, our data in *Drosophila* indicate that Hpo overexpression (Dcg > + vs. Dcg > Hpo) decreases lifespan, and Hpo depletion (Dcg > + vs. Dcg > Hpo‐RNAi) increases lifespan (Figure 1A). Although we observed changes in *C. elegans* lifespan and budding yeast growth, we do not know the extent of cst‐1 or Ste20 overexpression in *C. elegans* or yeast. This difference could be due in part to the degree of Hpo depletion in RNAi lines and residual Hpo expression in these animals. Alternatively, the presence of different isoforms (Mst1 homologs), tissue specificity, or other orthologues that have not yet been discovered could contribute to this difference. Regardless of these possibilities, the functions of Mst‐1/C‐cst‐1 in *Drosophila* and *C. elegans* clearly differ, requiring further studies of relevant pathways.

Me31b/RCK is an ATP‐dependent RNA helicase that suppresses mRNA translation and promotes mRNA decay (Wang et al. 2017) (Nakamura et al. 2004) (Coller and Parker 2005). It is a core component of ribonucleoprotein complexes (RNPs) such as processing bodies (P‐bodies) and the RNA‐induced silencing complex (RISC), which are involved in mRNA decay and translation (Barbee et al. 2006; Wilhelm et al. 2005; Glasmacher et al. 2010). Interestingly, we demonstrated that Mst1‐mediated Me31b/Rck phosphorylation stabilizes *Akh*/*Gcg* mRNA (Figures 3 and 4). A previous study identified Amyloid precursor protein (*APP*) mRNA as a RCK/p54 target in the human neuroblastoma cell line SH‐SY5Y, which is elevated when RCK is overexpressed, suggesting a possible function for RCK in mRNA stabilization (Broytman et al. 2009). We also observed that Akh elevation induces lipolysis and increases lipid droplet diameter in a Me31b‐dependent manner (Figures 1 and 6). However, Me31b or Rck may not be the only RBPs phosphorylated by Hpo or Mst1 relevant to lipid metabolism and lifespan regulation. Although the molecular mechanisms by which lipid metabolism is connected to lifespan regulation are incompletely understood (Hansen et al. 2013), our findings suggest that this non‐canonical Hpo pathway promotes lipolysis, decreasing lifespan.

Lifespan regulation by the *Drosophila* Hippo pathway could be mediated by direct phosphorylation of its target proteins. Although the effect of the Hippo pathway on lifespan in *Drosophila* and *C. elegans* differs, FOXO is a potential target protein. Increased *dFoxo* mRNA abundance could support this pathway, or Hpo could potentially directly phosphorylate dFoxo protein. eIF4E is another target protein, which was recently identified to be phosphorylated by Hpo in humans (Min et al. 2017). However, poor conservation of phosphorylation sites on *Drosophila* eIF4E compromises the feasibility of testing this hypothesis, but additional serine/threonine sites could be identified in future studies. Although silencing or overexpressing eIF4E did not significantly alter *Drosophila* lifespan (Figure S4A), eIF4E depletion or overexpression after Hpo overexpression could potentially regulate lifespan. Moreover, Hpo‐mediated increases of *eIF4E* and *4EBP* mRNA levels (Figure S1C) suggest the involvement of the Hippo pathway in the transcription or decay of these target genes.

### 5.2 Hippo Pathway Regulation of Lipid Metabolism

The Hippo pathway controls metabolic processes in pathologies such as obesity, type 2 diabetes (T2D), non‐alcoholic fatty liver disease (NAFLD), and cardiovascular disorders (Ardestani et al. 2018). For example, Hippo pathway activation negatively regulates lipid metabolism. The Hippo core kinases MST and LATS suppress hepatic lipogenesis by inhibiting sterol regulatory element‐binding protein (SREBP), a master regulator of lipogenesis (Aylon et al. 2016). Similarly, liver‐specific MST1 knockout also results in increased hepatic lipid droplet accumulation and liver degeneration via stabilization of sirtuin 1 (SIRT1), an important regulator of hepatic lipid metabolism in the context of high‐fat diet (HFD) consumption (Geng et al. 2016). The newly identified metabolic effect of the Hippo kinase MST1 through a non‐canonical Hippo pathway has direct implications for potential regulation of hepatic lipid metabolism and tumorigenesis (Geng et al. 2016; Zhou et al. 2009).

In the present study, we first identified that the Hippo kinase hMST1/dHpo regulates lipid metabolism by stabilizing *Akh* mRNA. AKH, a functional equivalent of mammalian glucagon, is so named due to its stimulatory effect on TAG mobilization in migratory locusts (Mayer and Candy 1969). Akh is produced in and secreted from a specialized endocrine organ, the corpora cardiaca (CC), in *Drosophila* and acts on the fat body, promoting lipolysis‐dependent energy metabolism by binding Akhr, a G protein‐coupled receptor expressed on fat cells (Kim and Rulifson 2004). Both *Drosophila* Akh and mammalian glucagon signaling are transduced by the second messenger cAMP and its subsequent activation of protein kinase A (PKA), increasing lipolysis by activating adipose tri‐glycerol lipase (ATGL), which converts TAGs to DAGs (Park et al. 2002). Consequently, the accumulation or abundance of DAG causes protein kinase C (PKC) activation and insulin resistance, leading to defective lipid metabolism and accelerated aging (Finck and Hall 2015).

The present study identified the accumulation of DAG, but not FFAs, in *Drosophila* fat bodies overexpressing Hpo (Figure 1). Although we have observed the decline of TAG levels and the elevation of DAG levels after Hpo overexpression, we did not observe any dramatic changes in FFA levels. Considering that the level of mRNAs encoding monoacylglycerol lipases is upregulated in RNA‐seq analysis, it is highly expected to have an increase in FFA levels as well. We possibly interpret these results as the generation of FFA being compensated with further breakdown of FFA after Hpo overexpression. In addition to the conversion of TAG to DAG by increased ATGL, further studies are necessary to verify that increased HSL and MGL do not cause additional conversion to MAG and fatty acids (Figure S1D). However, our findings suggest that the activation of both canonical and non‐canonical Hippo signaling could impair lipolysis and enhance DAG accumulation, resulting in decreased lifespan. Consistently, DAG accumulation is increased and insulin sensitivity is impaired in HSL‐deficient mice, while muscle TAG accumulation is increased and insulin sensitivity is improved in the ATGL‐deficient mice (Haemmerle et al. 2006; Osuga et al. 2000).

Overexpression of *Drosophila* Akh induced both hyperglycemia and hyperlipidemia in *Drosophila* circulating blood, hemolymph (Lee and Park 2004). In addition, *Drosophila* neurons lacking Akh have reduced trehalose levels and are resistant to nutrient deprivation (Gáliková et al. 2015). Notably, the null mutation of Akhr in *Drosophila* impaired TAG mobilization compared with reversion mutation of Akhr flies (Bharucha et al. 2008), indicating that Akh and Akhr could promote the conversion of TAG to DAG. These suggest that Hpo overexpression induced the transition from TAG to DAG through the stabilization of Akh. However, there have been no reports on how alterations in Akh stability and secretion affect lifespan, although the roles of Akh‐Akhr signaling in lipid metabolism are well‐known. A recent study reported that a high‐fat diet (HFD) feeding, which decreases lifespan, elevated Akh transcript levels, and that null mutation of Akh extended the longevity of *Drosophila* compared to the control feeding (Liao et al. 2021). Considering the elevation of DAG levels and decline of TAG levels, Hpo overexpression may compel the organism to continuously mobilize and utilize energy reserves, which could stress the organism and accelerate aging.

Previous work by Huang et al. (2013) demonstrated that under starvation conditions, Hpo overexpression in the *Drosophila* fat body decreased lifespan, whereas expression of myc‐Yki (S168A) partially ameliorated these survival defects. This suggests that Yki/YAP/TAZ might also influence lifespan reduction due to Hpo overexpression under normal conditions. However, our current study emphasizes the importance of RNA metabolism over the canonical Hippo pathway mechanisms. This focus allows us to explore novel regulatory aspects contributing to the observed phenotypes. Hpo overexpression elevates the steady‐state levels of dap, the *Drosophila* homolog of mammalian p16, p21, and p27, as well as p53. These findings imply a significant role for Hpo in modulating senescence and potentially elevating cancer risk through these pathways. Our RNA‐seq results highlighted the elevation of senescence‐associated mRNAs, including *p16* and *p53*. A notable increase in γ‐H2AX foci in the context of Hpo overexpression reinforces the link between Hpo activity, cellular senescence, and potentially heightened cancer susceptibility.

Comprehensively, we revealed that Hpo overexpression enhanced the activity of Me31b/RCK to promote adipocyte differentiation and lipolysis and *Drosophila* aging. Our findings provide insights into a non‐canonical function of Hpo and contribute to a deeper understanding of lipid metabolism and aging.

## Author Contributions

The research was conceived and designed by E.Y., H.M., J.L., J.‐H.Y., and K.‐S.L. The majority of experiments were performed by E.Y., H.M., and J.L. Y.L.C., and K.‐W.M., performed RIP RT‐qPCR analysis. J.L. and L.A.C. performed the adipocyte differentiation assay. J.S.P. and B.O. performed lipidomics analyses. Y.‐S.H., Y.S.L., and S.‐J.K. performed 3 T3‐L1 differentiation after transduction of lentiviral constructs, and then analyzed the results. J.H.C. performed protein purification and structure prediction. J.R.B. performed yeast lifespan analysis. J.P. and M.K. performed yeast and *C. elegans* lifespan assays. S.‐U.K. and S.Y.Y. performed RNA‐seq and Gene Ontology analysis. E.‐S.K. performed *C. elegans* lifespan assays. K.‐P.L. collected mouse muscle samples. E.Y., H.M., C.H.S., S.K., J.L., J.‐H.Y., and K.‐S.L. analyzed the data and wrote the manuscript.

## Conflicts of Interest

The authors declare no conflicts of interest.

## Supporting information


**Figure S1.** Whole‐body Hippo overexpression decreases lifespan.
**Figure S2.** Cst1 overexpression in *C. elegans* increases lifespan and ste20 deletion decreases lifespan in *S. cerevisiae*.
**Figure S3.** Lipid profiling and mouse adipocyte differentiation after treatment with the Mst1 inhibitor Xmp‐mu‐1, ROS scavenger N‐acetyl‐L‐cysteine, or CYP2E1 inhibitor Chlormethiazole.
**Figure S4.** eIF4E depletion decreases *Drosophila* lifespan.
**Figure S5.** RBPs targeted by cst‐1 and Ste20 are required for lifespan regulation in *C. elegans* and *S. cerevisiae*.
**Figure S6.** Lipid profiling of lysates from Dcg > +, Dcg > Hpo, Dcg > Hpo/me31b RNAi, and Dcg > Hpo/Dcp2 RNAi.

**Table S1.** Primer information.

**Table S2.** Drosophila RNA‐seq data.

**Table S3.** Gene Ontology.

## Data Availability

The RNA‐seq data have been deposited in the GEO database under accession code GSE201903. The data that support the findings of this study are available from the corresponding author upon reasonable request.
