# Supplementary material for: Phosphorylation of an RNA‐Binding Protein Rck/Me31b by Hippo Is Essential for Adipose Tissue Aging
Source: Aging Cell. 2025 Mar 11;24(6):e70022. doi: 10.1111/acel.70022 (PMC12151888; doi:10.1111/acel.70022)
Supplement: Supplementary file 1 — Figure S1. Whole‐body Hippo overexpression decreases lifespan. Figure S2. Cst1 overexpression in C. elegans increases lifespan and ste20 deletion decreases lifespan in S. cerevisiae. Figure S3. Lipid profiling and mouse adipocyte differentiation after treatment with the Mst1 inhibitor Xmp‐mu‐1, ROS scavenger N‐acetyl‐L‐cysteine, or CYP2E1 inhibitor Chlormethiazole. Figure S4. eIF4E depletion decreases Drosophila lifespan. Figure S5. RBPs targeted by cst‐1 and Ste20 are required for lifespan regulation in C. elegans and S. cerevisiae. Figure S6. Lipid profiling of lysates from Dcg > +, Dcg > Hpo, Dcg > Hpo/me31b RNAi, and Dcg > Hpo/Dcp2 RNAi. [file ACEL-24-e70022-s002.pdf]

Figure S1.

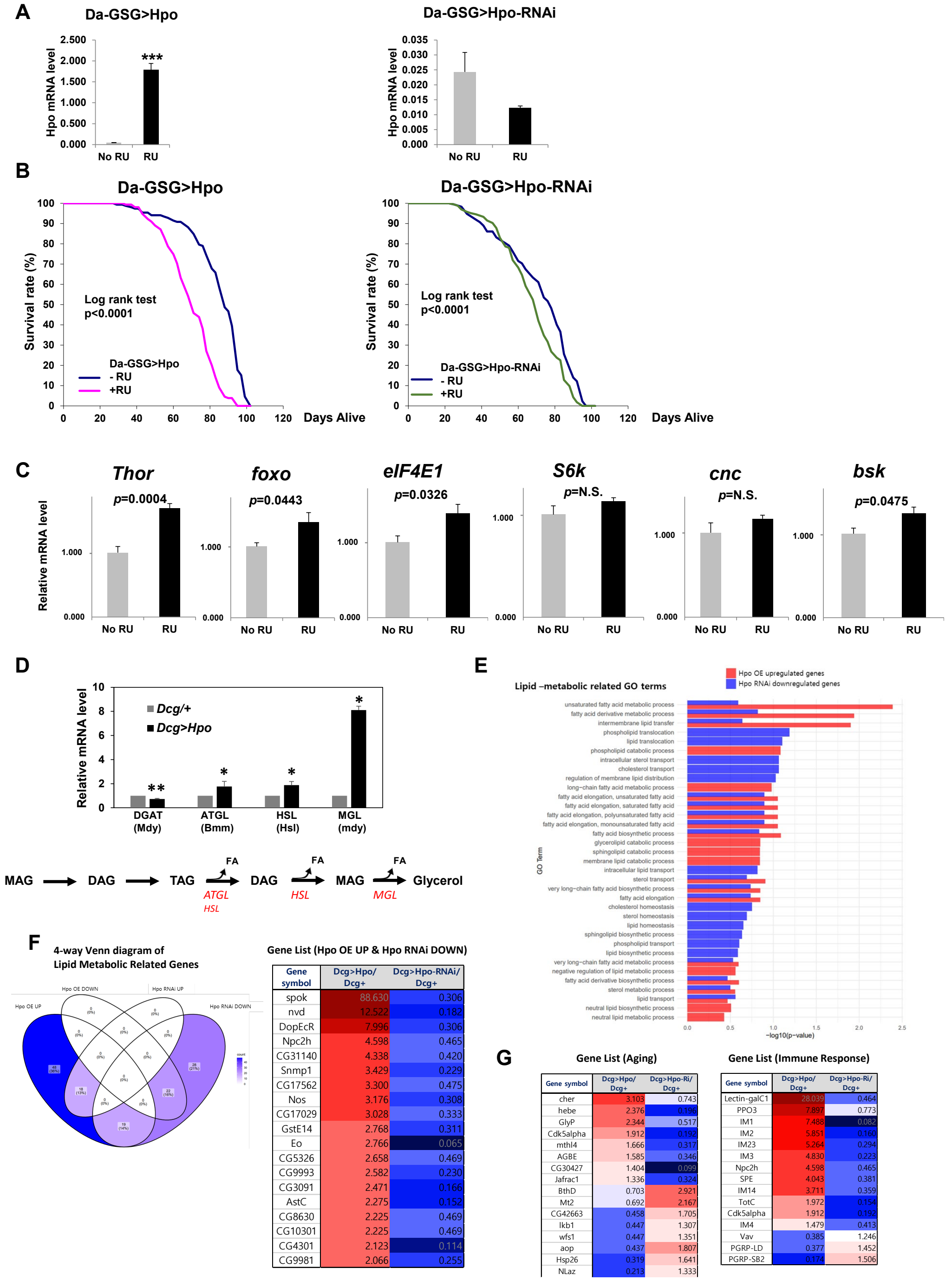

Gene List (Hpo OE UP & Hpo RNAi DOWN)

Gene symbol	Dcg>Hpo/ Dcg+	Dcg>Hpo-RNAi/ Dcg+
spok	88.630	0.306
nvd	12.522	0.182
DopEcR	7.996	0.306
Npc2h	4.598	0.465
CG31140	4.338	0.420
Snmp1	3.429	0.229
CG17562	3.300	0.475
Nos	3.176	0.308
CG17029	3.028	0.333
GstE14	2.768	0.311
Eo	2.766	0.065
CG5326	2.658	0.469
CG9993	2.582	0.230
CG3091	2.471	0.166
AstC	2.275	0.152
CG8630	2.225	0.469
CG10301	2.225	0.469
CG4301	2.123	0.114
CG9981	2.066	0.255

Gene List (Aging)

Gene symbol	Dcg>Hpo/ Dcg+	Dcg>Hpo-Ri/ Dcg+
cher	3.103	0.743
hebe	2.376	0.196
GlyP	2.344	0.517
Cdk5alpha	1.912	0.192
mthl4	1.666	0.317
AGBE	1.585	0.346
CG30427	1.404	0.099
Jafrac1	1.336	0.324
BthD	0.703	2.921
Mt2	0.692	2.167
CG42663	0.458	1.705
Ikb1	0.447	1.307
wfs1	0.447	1.351
aop	0.437	1.807
Hsp26	0.319	1.641
NLaz	0.213	1.333

Gene List (Immune Response)

Gene symbol	Dcg>Hpo/ Dcg+	Dcg>Hpo-Ri/ Dcg+
Lectin-galC1	28.039	0.464
PPO3	7.897	0.773
IM1	7.488	0.082
IM2	5.851	0.160
IM23	5.264	0.294
IM3	4.830	0.223
Npc2h	4.598	0.465
SPE	4.043	0.381
IM14	3.711	0.359
TotC	1.972	0.154
Cdk5alpha	1.912	0.192
IM4	1.479	0.413
Vav	0.385	1.246
PGRP-LD	0.377	1.452
PGRP-SB2	0.174	1.506

Figure S1: Whole-body Hippo overexpression decreases lifespan. (A) RT-qPCR measurement of *Hpo* mRNA in total RNA purified from head homogenates of DA-Gal4>Hpo (*left*) and DA-Gal4>Hpo-RNAi (*right*) animals with or without RU feeding. N = 5, ***p < 0.001 from Student's *t*-test. **(B)** Survivorship analysis of wild-type (DA-GSG/+, -RU) and Hippo-overexpressing (Da-GSG>Hpo, +RU) *D. melanogaster* (*left*). Survivorship analysis of wild-type (DA-GSG/+, -RU) and Hippo RNAi (DA-GSG>Hpo-RNAi, + RU) *D. melanogaster* (*right*). N = 152 (DA-GSG/+, -RU, *left*), 155 (Da-GSG>Hpo, +RU, *left*), 172 (DA-GSG/+, -RU), 164 (DA-GSG>Hpo-RNAi, + RU). **(C)** RT-qPCR measurement of *Thor*, *foxo*, *eIF4E1*, *S6k*, *cnc*, and *bsk* mRNA levels in total RNA purified from head homogenates of Da-GSG>Hpo animals with or without RU feeding. N = 5. **(D)** RT-qPCR measurement of *mdy*, *bmm*, *hsl* and *mdy* mRNA levels using total RNA isolated from fat bodies of third instar larvae in Dcg/+ and Dcg>Hpo animals. Data are expressed as mean ± SD of three independent experiments. N = 3, **p < 0.01, *p < 0.05 from Student's *t*-test. **(E)** Lipid metabolism-related Gene Ontology terms for both Hpo overexpression and Hpo knockdown data. Significantly enriched terms (*p*-value < 0.05) for genes upregulated in Hpo overexpression and genes downregulated in Hpo knockdown were shown. **(F)** 4-way Venn diagram of lipid metabolism-related genes and the list of overlapping genes that are upregulated in Hpo OE while being downregulated in Hpo knockdown. **(G)** Gene lists of aging- and immune response-related genes that are oppositely fluctuated under Hpo overexpression and knockdown conditions.

Figure S2.

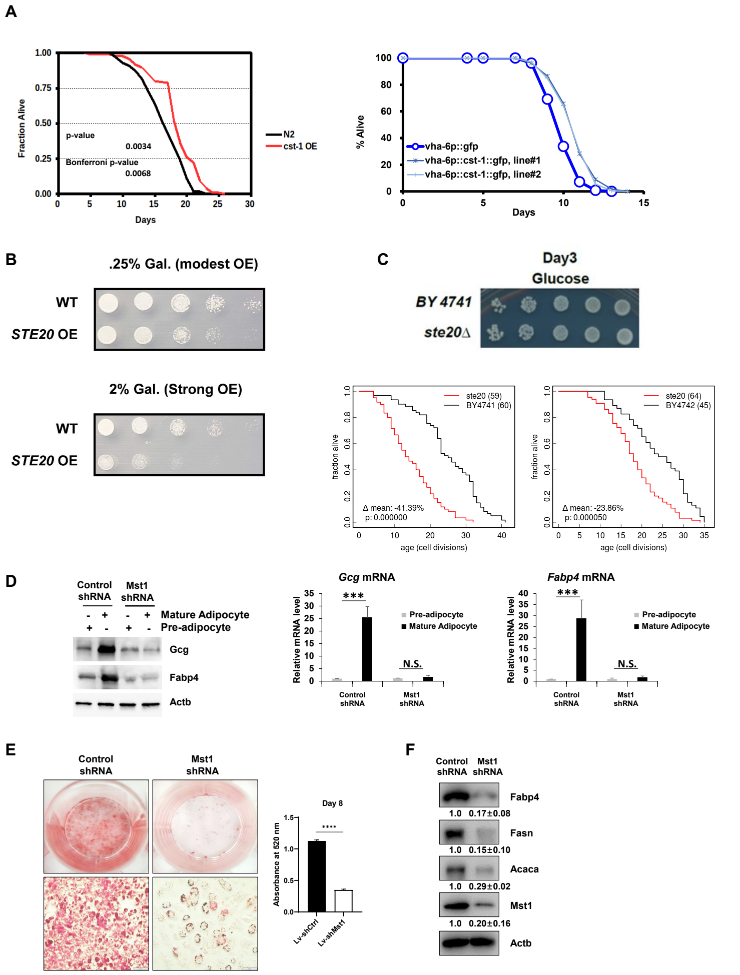

Figure S2: Cst1 overexpression in *C. elegans* increases lifespan and ste20 deletion decreases lifespan in *S. cerevisiae*. (A) Survivorship analysis of wild-type (N2 control) and cst-1-overexpressing *C. elegans*. ($p < 0.01$, log rank test; $p < 0.01$, Bonferroni p-value) (*left*). Survivorship analysis of wild-type (vha-6p::gfp) and intestine-specific Cst-overexpressing *C. elegans* (vha-6p::cst-1::gfp line 1 and 2) (*right*). ($P<0.001$, log rank test) N = 237 (*right*). **(B)** BY4741 *S. cerevisiae* expressing empty vector (pRS416) or GAL1-STE20 (pDH166) were serially diluted (1/5) on 1.75% Sucrose/0.25% Galactose media (*top*) or 2% Galactose media to modestly or strongly overexpress STE20 (*bottom*). Yeast strains were cultured continuously in 2% Galactose media and spotted in each dilution. All images represent 2 days of growth and are indicative of 3 biological replicates. Transformants did not differ from glucose media (non-inducing conditions; data not shown). **(C)** Growth of wild-type (BY4741) and ste20Δ strains 3 days after serial dilution (*top*). Survivorship analysis of wild-type (BY4741 and 4742) and ste20Δ strains of *S. cerevisiae*. ($p<0.001$, log rank test) (*bottom*). **(D)** mRNA (RT-qPCR, *right*) and protein (Western blot, *left*) levels of *Fabp4*, *Gcg*, and *Actb* mRNAs and proteins from pre-adipocytes (Day 0) and mature adipocytes (Day 8) after transfection of the Mst1 shRNA or control. Values are expressed as mean ± standard deviation (S.D.) of three independent experiments. ($p < 0.001$, Student's *t*-test). N = 3, ***$p < 0.001$, N.S., not significant from Student's *t*-test. **(E)** Oil Red O staining of 3T3-L1 cells infected with Lv-shMst1-SC (control) or Lv-shMst1 plasmid followed by adipogenic induction for day 8 (*left*). Relative lipid staining was quantitated by using ELISA at 520 nm ($p < 0.001$, Student's *t*-test) (*right*). N = 3, ****$p < 0.001$. **(F)** Protein (Western blot) levels of Fabp4, Fasn, Acaca, Mst1, and Actb from mature adipocytes (Day 8) after infection of infected with Lv-shMst1-SC (control) or Lv-shMst1 plasmid. The band intensity is quantitated as mean ± SD of three independent experiments. (****$p < 0.001$, Student's *t*-test).

Figure S3.

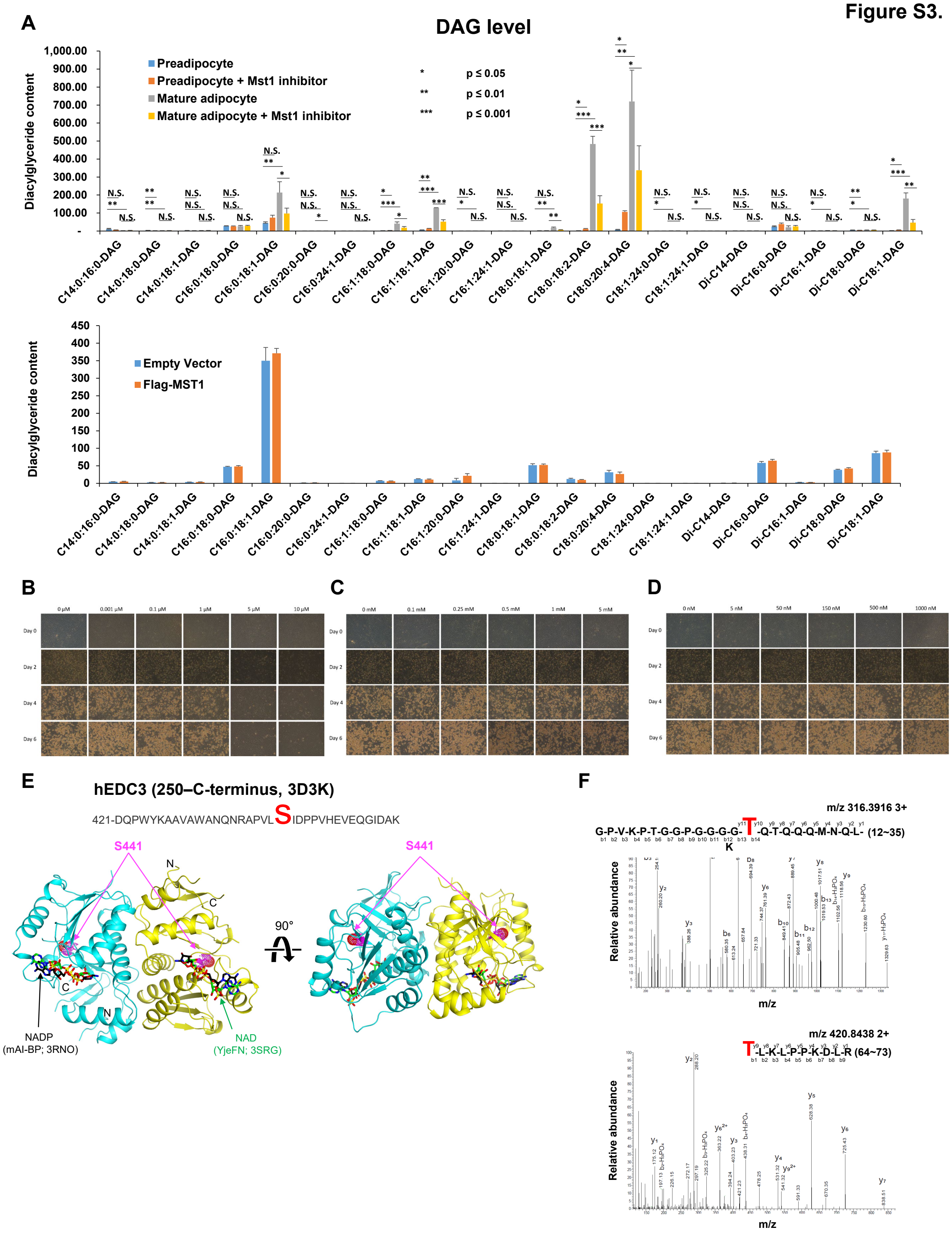

Figure S3: Lipid profiling and mouse adipocyte differentiation after treatment with the Mst1 inhibitor Xmp-mu-1, ROS scavenger N-acetyl-L-cysteine, or CYP2E1 inhibitor Chlormethiazole. (A) Measurement of DAG species in pre-adipocytes and mature adipocytes with or without Mst1 inhibitor treatment (*top*) as well as with transfection of empty or Flag-Mst1 plasmid (*bottom*). N = 3, ***$p < 0.001$, **$p < 0.01$, *$p < 0.05$, N.S., not significant from Student's *t*-test. **(B, C, and D)** Phase contrast images of differentiated primary mouse adipocytes on days 0, 2, 4, and 6 after Mst1 inhibitor (Xmp-mu-1) treatment, (*B*) ROS scavenger (N-Acetyl-L-Cysteine) treatment, (*C*) CYP2E1 inhibitor (Chlormethiazole) treatment, or (*D*) Vehicle (DMSO) on day 0 at the specified concentrations. **(E)** Crystal structure of human EDC3 (3D3K) with MST1-phosphorylated residues highlighted. **(F)** Mass spectra of peptides from Rck phosphorylated by MST1. After performing an *in vitro* phosphorylation reaction of MST1 with Rck, mass spectrometric analysis was performed. Product b- and y-ions are indicated in the peptide sequence and mass spectra. Mass spectra demonstrated that the Thr-36 and Thr-75 residues of Rck were phosphorylated by MST1.

Figure S4.

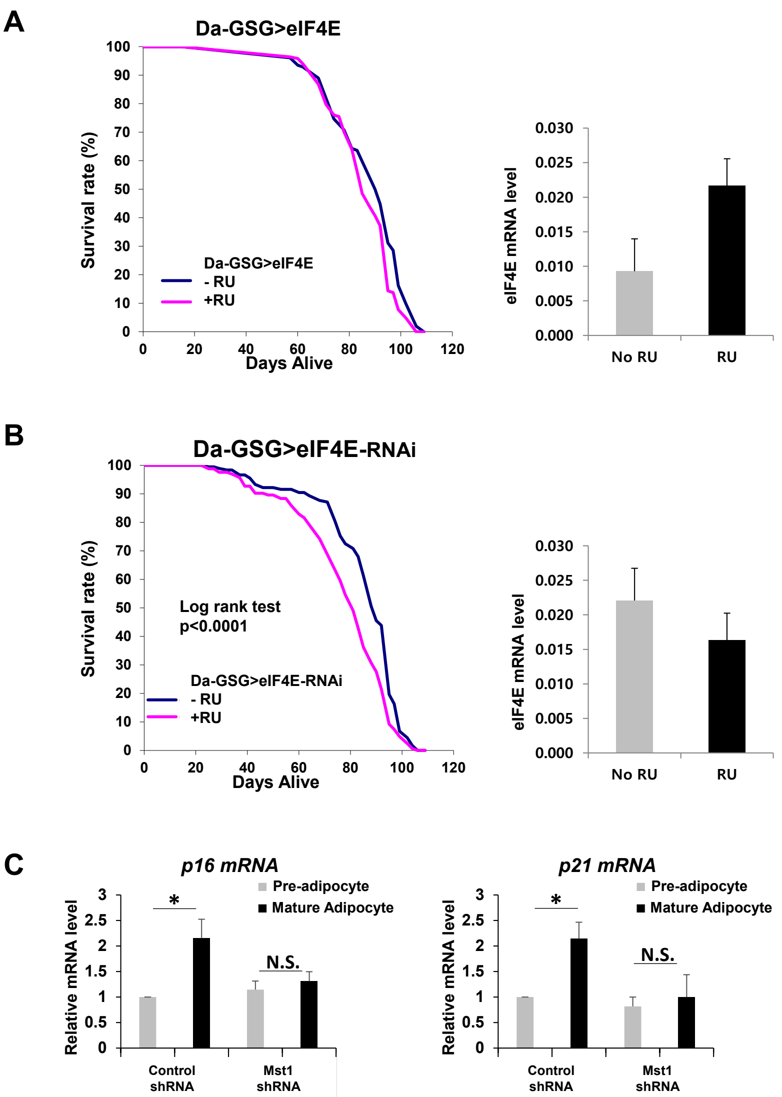

Figure S4: eIF4E depletion decreases *Drosophila* lifespan. (A) Survivorship analysis of wild-type (DA-GSG>eIF4E, -RU) and eIF4E-overexpressing (DA-GSG>eIF4E, +RU) *D. melanogaster* (*left*), and RT-qPCR measurement of *eIF4E* mRNA levels in total RNAs purified from head homogenates (*right*). N (*left*) = 154 (DA-GSG>eIF4E, -RU), 167 (DA-GSG>eIF4E, +RU). N (*right*) = 5. **(B)** Survivorship analysis of wild-type (DA-GSG>eIF4E-RNAi, -RU) and eIF4E RNAi (DA-GSG>eIF4E-RNAi, +RU) *D. melanogaster* (*left*) ($p < 0.0001$, log rank test), and RT-qPCR measurement of *eIF4E* mRNA levels in total RNAs purified from head homogenates (*right*). N = 178 (DA-GSG>eIF4E-RNAi, -RU), 163 (DA-GSG>eIF4E-RNAi, +RU). N = 5. **(C)** mRNA (RT-qPCR) levels of *p16* and *p21* mRNAs from pre-adipocytes (Day 0) and mature adipocytes (Day 8) after transfection of the Mst1 shRNA or control. Values are expressed as mean ± SD of three independent experiments. ($p < 0.001$, Student's *t*-test). N = 3, *p < 0.05, N.S., not significant from Student's *t*-test.

Figure S5.

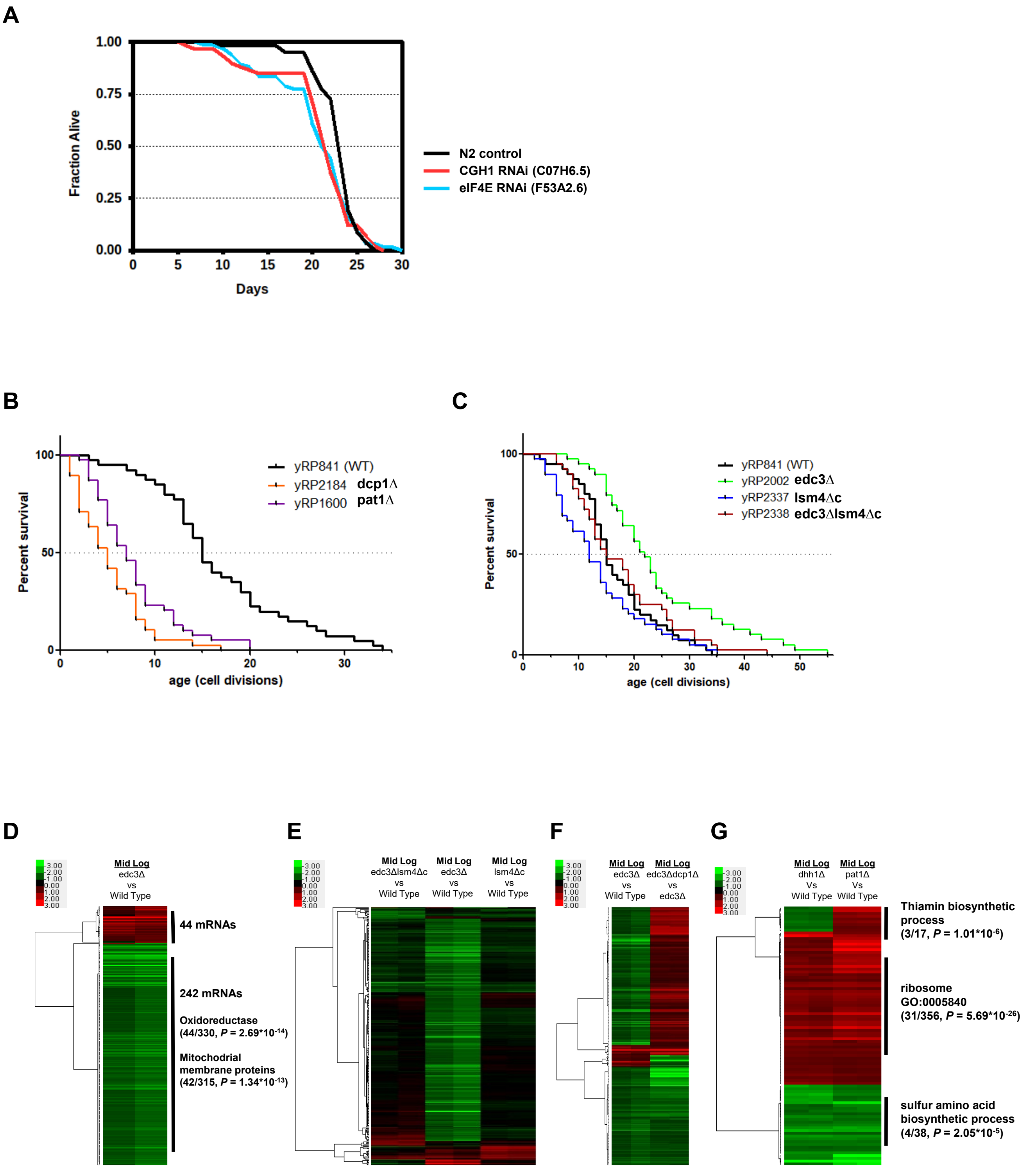

Figure S5: RBPs targeted by cst-1 and Ste20 are required for lifespan regulation in *C. elegans* and *S. cerevisiae*. (A) Survivorship analysis of wild-type (N2 control), *cgh-1* RNAi, and *eIF4E1* RNAi *C. elegans*. **(B)** Survivorship analysis of wild-type (yRP841), dcp1Δ, and pat1Δ *S. cerevisiae* strains. **(C)** Survivorship analysis of wild-type (yRP841), edc3Δ, lsm4Δc, and edc3Δlsm4Δc *S. cerevisiae* strains. **(D-G)** cDNA microarray analysis of total RNAs purified from wild-type, edc3Δ (*D*), lsm4Δc, edc3Δlsm4Δc (*E*), edc3Δdcp1Δ (*F*), pat1Δ, and dhh1Δ (*G*) *S. cerevisiae* strains in mid-log growth conditions.

Figure S6.

A

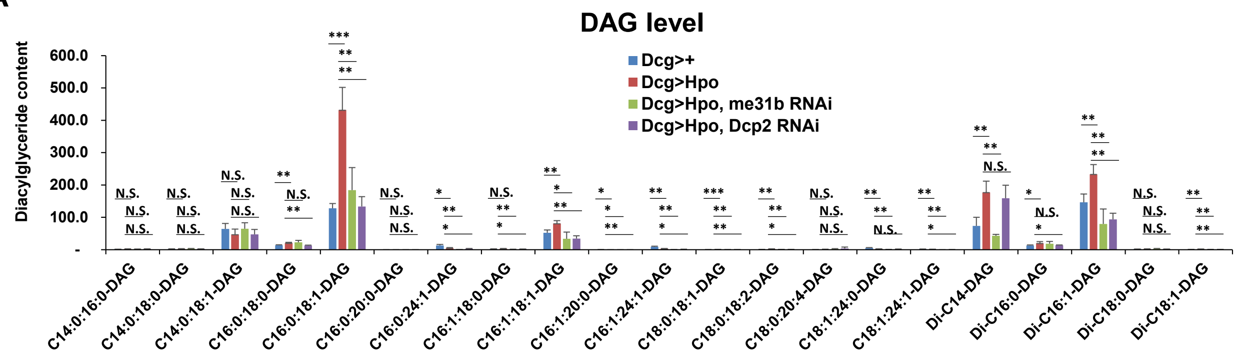

B

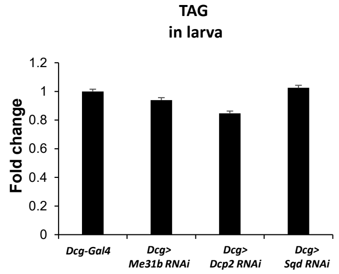

C

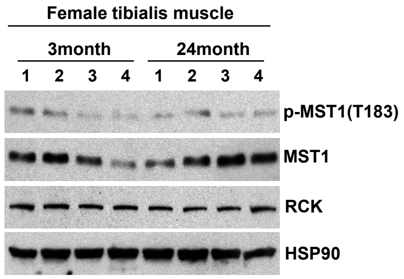

Figure S6: Lipid profiling of lysates from Dcg>+, Dcg>Hpo, Dcg>Hpo/me31b RNAi, and Dcg>Hpo/Dcp2 RNAi. (A) Profiling of DAG species in homogenates from Dcg>+, Dcg>Hpo, Dcg>Hpo/me31b RNAi, and Dcg>Hpo/Dcp2 RNAi *Drosophila*. N = 3, ***$p < 0.001$, **$p < 0.01$, *$p < 0.05$, N.S., not significant from Student's *t*-test. **(B)** Relative TAG levels were measured by TAG assay in Dcg>+, Dcg>Me31b RNAi, Dcg>Dcp2 RNAi, and Dcg>Sqd RNAi *Drosophila* third instar larvae and Dcg>+, Dcg>Hpo and Dcg>Hpo, me31B RNAi 5-day-old *Drosophila* adult males. N = 3. **(C)** Western blot analysis of p-MST1, MST1, RCK, and Heat Shock Protein 90 (HSP90) in young (3-month-old) and aged (24-month-old) female mouse tibialis muscles.
